# Injectable hybrid inorganic nanoscaffold as rapid stem cell assembly template for cartilage repair

**DOI:** 10.1093/nsr/nwac037

**Published:** 2022-02-28

**Authors:** Shenqiang Wang, Letao Yang, Bolei Cai, Fuwei Liu, Yannan Hou, Hua Zheng, Fang Cheng, Hepeng Zhang, Le Wang, Xiaoyi Wang, Qianxin Lv, Liang Kong, Ki-Bum Lee, Qiuyu Zhang

**Affiliations:** Key Laboratory of Special Functional and Smart Polymer Materials of Ministry of Industry and Information Technology, School of Chemistry and Chemical Engineering, Northwestern Polytechnical University, Xi’an 710129, China; Xi’an Key Laboratory of Functional Organic Porous Materials, School of Chemistry and Chemical Engineering, Northwestern Polytechnical University, Xi’an 710072, China; Department of Chemistry and Chemical Biology, Rutgers University, Piscataway, NJ 08854, USA; Department of Chemistry and Chemical Biology, Rutgers University, Piscataway, NJ 08854, USA; Department of Biomedical Engineering, Columbia University, New York, NY 10032, USA; State Key Laboratory of Military Stomatology & National Clinical Research Center for Oral Diseases & Shaanxi Key Laboratory of Oral Diseases, Department of Oral and Maxillofacial Surgery, School of Stomatology, The Fourth Military Medical University, Xi’an 710032, China; State Key Laboratory of Military Stomatology & National Clinical Research Center for Oral Diseases & Shaanxi Key Laboratory of Oral Diseases, Department of Oral and Maxillofacial Surgery, School of Stomatology, The Fourth Military Medical University, Xi’an 710032, China; Department of Chemistry and Chemical Biology, Rutgers University, Piscataway, NJ 08854, USA; Key Laboratory of Special Functional and Smart Polymer Materials of Ministry of Industry and Information Technology, School of Chemistry and Chemical Engineering, Northwestern Polytechnical University, Xi’an 710129, China; Key Laboratory of Special Functional and Smart Polymer Materials of Ministry of Industry and Information Technology, School of Chemistry and Chemical Engineering, Northwestern Polytechnical University, Xi’an 710129, China; Key Laboratory of Special Functional and Smart Polymer Materials of Ministry of Industry and Information Technology, School of Chemistry and Chemical Engineering, Northwestern Polytechnical University, Xi’an 710129, China; Research & Development Institute of Northwestern Polytechnical University in Shenzhen, Shenzhen 518057, China; Xi’an Key Laboratory of Functional Organic Porous Materials, School of Chemistry and Chemical Engineering, Northwestern Polytechnical University, Xi’an 710072, China; State Key Laboratory of Military Stomatology & National Clinical Research Center for Oral Diseases & Shaanxi Key Laboratory of Oral Diseases, Department of Oral and Maxillofacial Surgery, School of Stomatology, The Fourth Military Medical University, Xi’an 710032, China; State Key Laboratory of Military Stomatology & National Clinical Research Center for Oral Diseases & Shaanxi Key Laboratory of Oral Diseases, Department of Oral and Maxillofacial Surgery, School of Stomatology, The Fourth Military Medical University, Xi’an 710032, China; State Key Laboratory of Military Stomatology & National Clinical Research Center for Oral Diseases & Shaanxi Key Laboratory of Oral Diseases, Department of Oral and Maxillofacial Surgery, School of Stomatology, The Fourth Military Medical University, Xi’an 710032, China; State Key Laboratory of Military Stomatology & National Clinical Research Center for Oral Diseases & Shaanxi Key Laboratory of Oral Diseases, Department of Oral and Maxillofacial Surgery, School of Stomatology, The Fourth Military Medical University, Xi’an 710032, China; Department of Chemistry and Chemical Biology, Rutgers University, Piscataway, NJ 08854, USA; Key Laboratory of Special Functional and Smart Polymer Materials of Ministry of Industry and Information Technology, School of Chemistry and Chemical Engineering, Northwestern Polytechnical University, Xi’an 710129, China; Research & Development Institute of Northwestern Polytechnical University in Shenzhen, Shenzhen 518057, China; Xi’an Key Laboratory of Functional Organic Porous Materials, School of Chemistry and Chemical Engineering, Northwestern Polytechnical University, Xi’an 710072, China

**Keywords:** injectable nanoscaffold, 3D cell culture, tissue engineering, cartilage repair, stem-cell therapy

## Abstract

Cartilage injuries are often devastating and most cannot be cured because of the intrinsically low regenerative capacity of cartilage tissues. Although stem-cell therapy has shown enormous potential for cartilage repair, the therapeutic outcome has been restricted by low survival rates and poor chondrocyte differentiation *in vivo*. Here, we report an injectable hybrid inorganic (IHI) nanoscaffold that facilitates fast assembly, enhances survival and regulates chondrogenic differentiation of stem cells. IHI nanoscaffolds that strongly bind to extracellular matrix (ECM) proteins assemble stem cells through synergistic 3D cell–cell and cell–matrix interactions, creating a favorable physical microenvironment for stem-cell survival and differentiation *in vitro* and *in vivo*. Additionally, chondrogenic factors can be loaded into nanoscaffolds with a high capacity, which allows deep, homogenous drug delivery into assembled 3D stem-cell-derived tissues for effective control over the soluble microenvironment of stem cells. The developed IHI nanoscaffolds that assemble with stem cells are injectable. They also scavenge reactive oxygen species and timely biodegrade for proper integration into injured cartilage tissues. Implantation of stem-cell-assembled IHI nanoscaffolds into injured cartilage results in accelerated tissue regeneration and functional recovery. By establishing our IHI nanoscaffold-templated 3D stem-cell assembly method, we provide a promising approach to better overcoming the inhibitory microenvironment associated with cartilage injuries and to advance current stem-cell-based tissue engineering.

## Teaser

They developed a 3D injectable hybrid inorganic (3D-IHI) nanoscaffold-based approach that significantly accelerated cartilage regeneration in a rabbit critical defect model by improving stem cell survival and chondrogenesis.

## INTRODUCTION

Injuries to articular cartilage and meniscus, especially those that result in critically sized cartilage defects, are often debilitating and can cause loss of joint functions at late stages [1]. However, current surgical approaches for cartilage injuries have led to limited regeneration and mostly target pain relief in the short term [2]. In this regard, a stem-cell-based tissue-engineering approach has recently shown great potential to rapidly restore injured cartilage tissues at early stages [3,4]. For example, stem cells that are transplanted successfully initially secrete trophic factors to reduce inflammation at sites of cartilage injuries and then differentiate into cartilage cells (e.g. chondrocytes) for functional restoration [5]. Considering their huge therapeutic potential for cartilage repair, several mesenchymal stem cell (MSC)-based treatments have recently entered clinical trials with promising results [6,7]. Nevertheless, there are critical barriers that remain to be overcome before the therapeutic potential of stem-cell therapies can be realized. First, due to the prevalence of oxidative stress and inflammation in the microenvironment of injury sites, stem cells frequently undergo apoptosis after injection [8,9]. Second, limited control over chondrogenic differentiation of stem cells *in vivo* often compromises regenerative outcomes [10–12].

To address these challenges, both scaffold- and scaffold-free tissue-engineering approaches have been developed to advance stem-cell therapies by providing mechanical support, delivering growth factors, modulating immune responses and facilitating integration of stem cells *in vivo* [13,14]. Despite their clear advantages, conventional tissue-engineering approaches for cartilage repair have also been hampered by several hurdles. For instance, microporous scaffolds that improve chondrogenesis of stem cells *in vitro* are often non-injectable, requiring invasive surgery procedures for implantation [2,15]. Hydrogel-based stem-cell grafts have a wide range of tunable mechanical properties and are generally injectable [16]. However, cell–cell interactions and 3D cell condensation, which are crucial for chondrogenesis and cartilage regeneration, can be hindered in hydrogel-based stem-cell culture due to the cross-linked hydrogel network [17–20]. In this regard, scaffold-free tissue-engineering technologies, such as 3D stem-cell spheroids and 2D cell sheets assembled from stem cells, have enormous advantages because they effectively preserve cell–cell interactions during cartilage regeneration and avoid side effects from exogenous scaffolding materials *in vivo* [21]. Nevertheless, current scaffold-free tissue-engineering approaches based on 3D stem-cell assemblies are still impeded by barriers including limited drug diffusion, the formation of a necrotic core, uncontrollable differentiation inside the 3D-assembled stem cells and the reduced capability to modulate the inflammatory and inhibitory microenvironment at tissue injury sites compared with scaffold-based approaches [22–24]. Additionally, current scaffold-free stem-cell assembly approaches, including spinner flask, liquid overlay and hanging-drop methods, can take days or even weeks to generate well-defined 3D spheroids, which may further delay treatment of cartilage injuries at acute phases [25]. Therefore, both scaffold- and scaffold-free tissue-engineering approaches have not harnessed the full therapeutic potential of stem cells for treatment of cartilage injuries. Thus, advancing transplantation strategies to achieve high survival rates and efficient chondrogenic differentiation of stem cells *in vivo* are urgently required.

**Figure 1.**
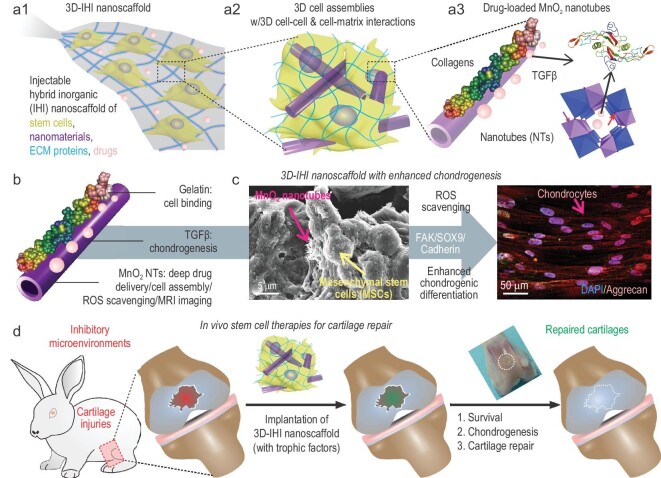
Enhanced treatment of cartilage injuries using IHI nanoscaffold-templated 3D stem-cell assembly. (a1) A schematic illustration of the 3D TGFβ–BMSC–IHI nanoscaffold. (a2) The 3D-IHI nanoscaffold enabled both cell–cell interaction and cell–matrix interaction, which favored the chondrogenic differentiation of BMSC. (a3) The hollow structure of MnO_2_ NTs were beneficial to load chondrogenic drugs/growth factors. (b) Schematic illustration of gelatin-coated and TGF-β3-loaded MnO_2_ NTs. (c) The FESEM image indicated that most of the BMSCs form contacts with other cells and the 1D fibril-like structures, which is similar to the structures of natural tissues. The enhanced chondrogenesis of BMSC was confirmed by the immunostaining of chondrogenic protein (Aggrecan). (d) By remodeling the oxidative microenvironment, enhancing the cell viability and chondrogenesis of transplanted cells, cartilage regeneration could be finally achieved.

To this end, we developed a 3D-injectable hybrid inorganic (IHI) nanoscaffold-based approach that combines the advantages of scaffold- and scaffold-free tissue engineering to enhance stem-cell-based treatment of cartilage injuries (Fig. 1). Remarkably, the 3D-IHI nanoscaffold improves survival and chondrogenic differentiation of stem cells by (i) rapidly assembling stem cells into 3D tissues with controllable cell–cell interactions; (ii) demonstrating high affinity for chondrogenic extracellular matrix (ECM) proteins to enhance 3D cell–matrix interactions; (iii) scavenging reactive oxygen species (ROS) and suppressing the inflammatory microenvironment by a unique MnO_2_ composition; and (iv) homogeneously delivering chondrogenic factors throughout 3D-assembled stem cells. As a proof-of-concept demonstration of 3D-IHI nanoscaffold-based stem-cell culture and cartilage differentiation, 1D biodegradable MnO_2_ nanotubes (NTs) coated with L-Arginyl-Glycyl-L-Aspartic acid (RGD)-rich gelatin were used to template the 3D assembly of stem cells. Specifically, unlike the conventional hydrogel-based covalent cross-linking mechanism to create 3D gel structures, IHI nanoscaffolds assemble stem cells through strong interactions among MnO_2_ NTs, gelatin and cells, which form 3D assembled structures (Fig. 1a and Table S1). In our study, MnO_2_ nanomaterials were chosen based on their robust biodegradability by bioreductants (e.g. vitamin C) secreted by endogenous cells, biocompatibility and ability to catalyse depletion of ROS and potential for magnetic resonance imaging (MRI) based on previous reports from us and others [26]. Meanwhile, 1D MnO_2_ NTs were selected because of their high surface area for drug (e.g. TGF-β) loading and protein binding, as well as their mimicry of 1D collagen fibrils in the cartilage ECM (Fig. 1b) [27]. Through optimization of 3D cell–cell and cell–matrix signaling in stem cells using our 3D-IHI nanoscaffold-templated assembly, we successfully derived chondrocyte-like cells from MSCs. Incorporation of IHI nanoscaffold-mediated deep, homogeneous drug delivery further enhanced chondrogenic differentiation *in vitro* (Fig. 1c). Notably, injection of stem-cell-assembled 3D-IHI nanoscaffolds into injured cartilage tissues in a rabbit critical defect model significantly reduced inflammation and improved stem-cell survival and chondrogenesis, thereby promoting cartilage regeneration and functional recovery in the long term (Fig. 1d). By developing our 3D-IHI nanoscaffold-templated stem-cell assembly, we provide a promising approach to advance current stem-cell therapies and improve the outcomes of cartilage-tissue engineering.

## RESULTS AND DISCUSSION

**Figure 2.**
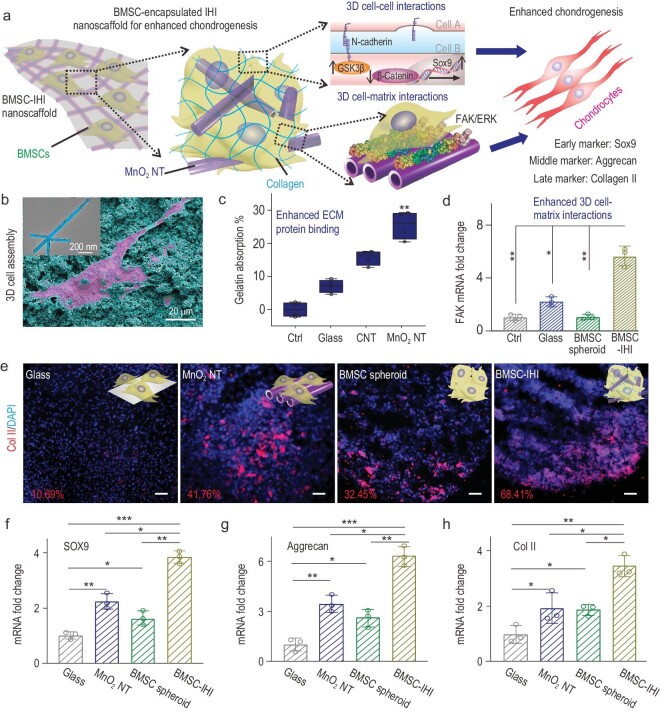
Creating the 3D-IHI nanoscaffold using biodegradable nanomaterials. (a) A schematic diagram showing that the 3D-IHI nanoscaffold could enhance chondrogenic differentiation of BMSC through a synergy between N-cadherin and FAK-mediated pathways. (b) The strong interactions between MnO_2_ NTs and functional groups commonly existing in ECM proteins effectively supported cell attachment as demonstrated via SEM image. The inset TEM image reveals the cubic hollow structure of the MnO_2_ NT (the red color indicates the BMSC, while the blue color indicates the MnO_2_ NTs). (c) Bicinchoninic acid assay indicates the enhanced absorption toward gelatin from MnO_2_ NT compared to control groups. (d) The MnO_2_ NT-templated assembly method significantly enhanced cell–matrix interaction as demonstrated through the upregulated expression patterns of the FAK gene. (e) Representative immunostaining images showing the improved chondrogenesis of BMSC in the BMSC–IHI nanoscaffold group compared to the control groups. The numbers represent the type II collagen (Col II) staining positive cells counted through imageJ. Scale bar: 50 μm. (f)–(h) The expression of chondrogenic genes, including SOX9 (f), Aggrecan (g) and Col II (h), were characterized via qRT-PCR measurement. All data are presented as mean ± SD (*n* = 4). ^*^P < 0.05, ^**^P < 0.01, ^***^P < 0.001.

The scheme in Fig. 2a illustrates the structure and assembly of our 3D-IHI nanoscaffold. Specifically, we synthesized the targeted nanoscaffold by incubating stem cells with gelatin-coated MnO_2_ NTs (Fig. 2b and Fig. S1). Considering the high clinical potential of bone-marrow-derived mesenchymal stem cells (BMSCs), we showcased the generation of a BMSCs-incorporated 3D-IHI nanoscaffold (BMSC–IHI), which was not only soft, mechanically stretchable (Movie S1) and injectable (Movie S2), but also ensured a high density of cells during transplantation. Strong binding between MnO_2_ NTs and gelatin is supported by our previous simulation study and was directly confirmed by a bicinchoninic acid protein assay (Fig. 2b and c) [26,28]. We also proved our hypothesis by observing extremely rapid (<1 h) aggregation of cells during their incubation with gelatin-coated MnO_2_ NTs (Fig. S2). This is nearly an order higher of speed compared with conventional stem-cell spheroid and cell-laden gel-formation methods (e.g. hanging-drop method) [29]. Successful generation of our targeted 3D-IHI nanoscaffold with both 3D cell–cell and 3D cell–matrix interactions was also verified by field-emission scanning electron microscopy (FESEM), whereby most cells formed contacts with other cells and fibril-like ECM (Fig. S3a) [30]. As controls, BMSCs incubated in soluble gelatin-formulated media (control group) or cultured on 2D glasses deposited with gelatin-coated MnO_2_ NTs (MnO_2_ NT group) did not show rapid 3D assembly of stem cells, which supported the essential role of MnO_2_ NTs in templating the nanoscaffold formation (Fig. S3b and c). Although high densities of BMSCs eventually settled down and adhered to each other after prolonged incubation in medium-alone conditions without NTs, the assembled cells were similar to 2D cultures and lacked the 3D interactions with surrounding ECMs and cells (Fig. S3d). Additionally, we observed significantly different expression patterns of genes related to cell–cell interactions (e.g. N-cadherin) and cell–matrix interactions (e.g. focal adhesion kinase, FAK) in the 3D nanoscaffold group compared with the control conditions, which indicated potential enhancement of downstream chondrogenic pathways (Figs 2d and S4a). Thus, we confirmed successful generation of a 3D-IHI nanoscaffold that encompassed both 3D cell–cell and cell–matrix interactions through a MnO_2_ nanotube-templated method.

In nature, cartilage is an avascular tissue composed of chondrocytes and ECM proteins, such as collagen and chondroitin sulfates [30]. Therefore, both cell–cell and cell–matrix interactions play crucial roles in regulating the chondrogenesis of stem cells as well as the activities of differentiated chondrocytes [31,32]. We hypothesized that simultaneous modulation of the cell–cell and cell–matrix interactions in our 3D-IHI nanoscaffold would effectively enhance chondrogenesis through a synergy between the Wingless-related integration site (Wnt) and FAK-mediated pathways. To test our hypothesis, we carried out four 14-day chondrogenic assays. Our hypothesis on enhanced chondrogenesis in the 3D-IHI nanoscaffold was verified by a significantly higher expression of a chondrogenic protein (Col II) and genes (Sox9, Aggrecan and Col II) in the BMSC–IHI nanoscaffold group as compared to the control conditions (Fig. 2e–h). Increased activation of Wnt inhibitory gene (glycogen synthase kinase-3β, GSK3β), FAK gene and ERK1/2 gene, and downregulation of Wnt-related gene (β-catenin) determined by quantitative real-time polymerase chain reaction (qRT-PCR) further supported our hypothesis on the synergistic effects of cell–cell and cell–matrix interactions in the 3D-IHI nanoscaffold on the chondrogenesis of BMSCs (Fig. S4b–f). Taken together, our 3D-IHI nanoscaffold alone effectively promoted the chondrogenesis of BMSCs by modulating cell–cell (e.g. Wnt) and cell–matrix (e.g. FAK and ERK)-related pathways.

**Figure 3.**
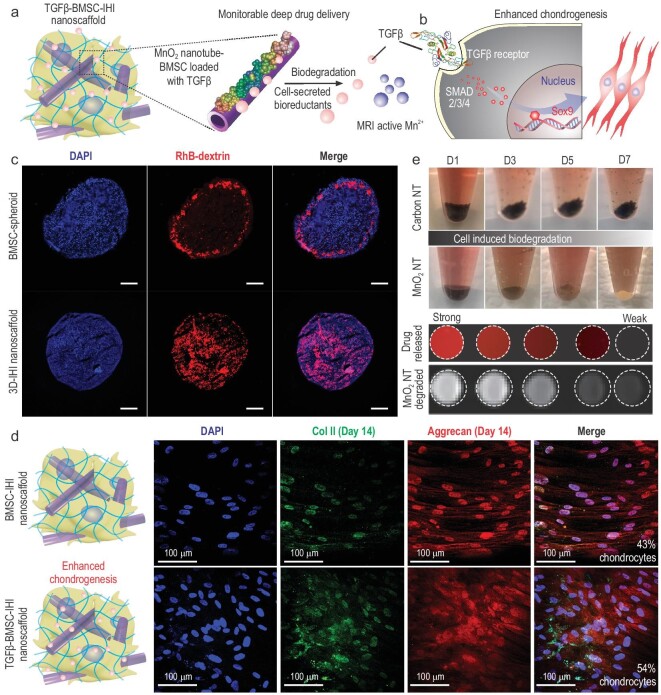
Deep delivery of soluble factors for enhancing stem-cell chondrogenesis. (a) Schematic diagram of drug releasing and monitoring in the TGFβ–BMSC–IHI nanoscaffold. (b) The effective and homogeneous delivery of TGF-β3 further improved the chondrogenic differentiation of BMSC through Smad pathways. (c) Fluorescence microscopic images demonstrating the deep and homogeneous delivery of model bio-macromolecular drug (Dex–RhB) in the MnO_2_ NT Dex–RhB-templated IHI nanoscaffold as compared to the control BMSC spheroids incubated with free Dex–RhB. Scale bar: 200 μm. (d) Immunostaining results on chondrogenic markers (Col II, labeled with green, Aggrecan, labeled with red) demonstrated significant enhancement of chondrogenesis of BMSC differentiated in the TGFβ–BMSC–IHI nanoscaffold compared to the BMSC–IHI nanoscaffold. (e) Time-dependent biodegradation of MnO_2_ NTs in cell culture without the addition of any external trigger. Carbon nanotube (CNT) was used as a negative control and no noticeable degradation was observed. The stoichiometrical release of T_1_ active Mn^2+^ enabled the monitoring of MnO_2_ NTs degradation and drug release, which was confirmed by a direct correlation between the amount of released drug (indicated by red fluorescence) and T_1_ MRI intensities detected from the nanoscaffold.

Next, the chondrogenic differentiation of stem cells in our 3D-IHI nanoscaffold was enhanced by deep and homogeneous delivery of chondrogenic factors. Deep delivery of transforming growth factor beta-3 (TGF-β3) was demonstrated as a proof of concept due to its broad clinical relevance [33]. Although the effects of TGF-β3-formulated medium on the chondrogenesis of MSC spheroids have been previously studied [34], efficient delivery of TGF-β3 to cells located at the inner core of 3D tissue constructs (e.g. spheroids) remains challenging due to the strong diffusion barrier in 3D cell-assembly systems [35]. In this regard, the 3D-IHI nanoscaffold-templated from MnO_2_ NTs with drugs loaded on both the surfaces and hollow pores of the MnO_2_ NTs (loading efficiency of 29.8%) achieved homogeneous distribution of TGF-β3 in 3D, thereby providing a promising means to overcome the drug-diffusion barrier (Figs 3a and b, and S5). The strong electrostatic and polar-π interactions between MnO_2_ nanomaterials and biomolecules contributed to the high drug-loading capacity [26,28]. To validate the drug distribution, we first loaded MnO_2_ NTs with a model bio-macromolecular drug [rhodamine B-labeled dextran (Dex)] with strong fluorescence and a similar molecular weight as TGF-β3 to monitor the drug distribution. Then, a 3D-IHI nanoscaffold was assembled from BMSCs and Rhodamine B (RhB)-loaded, gelatin-coated MnO_2_ nanotube (MnO_2_ NT Dex–RhB) using identical protocols (Fig. S6). As a control, BMSC spheroids were also incubated with RhB-supplemented medium for 1 h. We verified our hypothesis on MnO_2_ nanotube-mediated deep drug delivery by observing a more homogeneous drug-diffusion pattern in the MnO_2_ NT Dex–RhB-templated IHI nanoscaffold compared with the control BMSC spheroids incubated with free Dex–RhB (Fig. 3c). Importantly, when Dex–RhB was replaced with TGF-β3, the 3D-IHI nanoscaffold assembled from TGF-β3-loaded MnO_2_ NTs further enhanced chondrogenesis as compared to the control group (nanoscaffold assembled from MnO_2_ NTs only cultured in TGF-β3-supplemented medium). Specifically, immunostaining of chondrogenic markers confirmed 1.36-fold and 1.25-fold increases of Col II and Aggrecan, respectively, which suggested enhancement of chondrogenesis in our experimental group (Fig. 3d). Additionally, continuous upregulation of chondrogenic genes (Sox9, Aggrecan, Col II) were also observed throughout the 21-day differentiation experiment (Fig. S7). Taken together, our 3D-IHI nanoscaffold uniquely demonstrated deep drug delivery of chondrogenic factors that further upregulated chondrogenesis of BMSCs.

Our 3D-IHI nanoscaffold incorporated other unique material properties from MnO_2_ NTs desired for *in vivo* tissue-engineering applications. For example, a crucial factor that is often related to the inflammatory microenvironment and causes apoptosis of transplanted stem cells at sites of cartilage injuries has been attributed to over-production of ROS (e.g. H_2_O_2_) [36]. In this regard, our MnO_2_ NTs, known as a catalyst for scavenging ROS [37], could mitigate the hostile microenvironment at cartilage-injury sites and enhance the survival of stem cells. The enhanced scavenging of ROS and survival of stem cells in our IHI nanoscaffold were partially supported by our *in vitro* live/dead cell assay (Fig. S8). Additionally, MnO_2_ NTs in the 3D-IHI nanoscaffold were highly biocompatible, blood compatible, biodegradable and slowly released T_1_-weighted MRI-active Mn^2+^ as a contrast agent during biodegradation for potential *in vivo* imaging and monitoring of cartilage-injury sites (Figs 3e, S9 and S10). Lastly, we were able to demonstrate robust control over the size, length, shape and concentration of MnO_2_ nanomaterials, which allowed us to attain additional regulation of the biological functions of the 3D-IHI nanoscaffold (Figs S11–S13).

**Figure 4.**
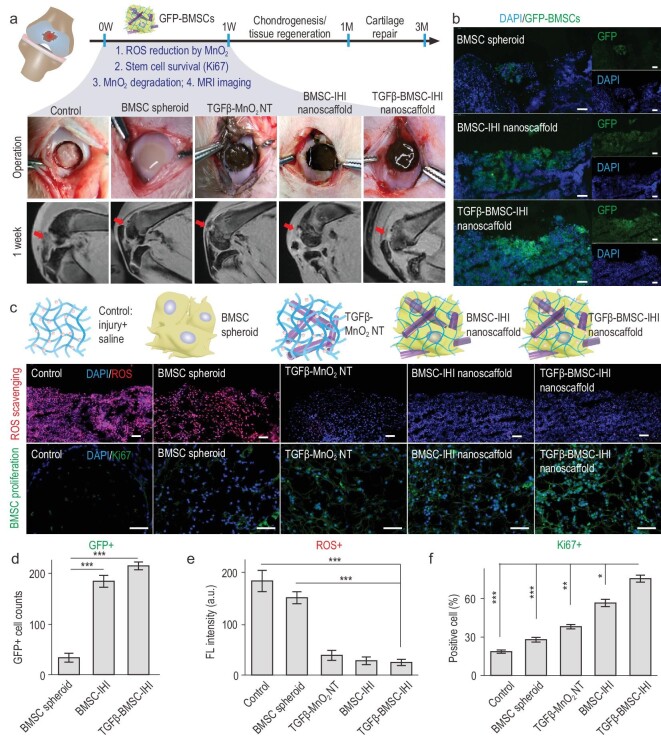
Improved stem-cell transplantation at cartilage-injury sites by 3D-IHI nanoscaffold. (a) Schematic diagram illustrating the surgical process and timeline of cartilage repair. Macroscopic views of the cartilage defects filled with TGFβ–BMSC–IHI nanoscaffold and the controls. The degradation of MnO_2_ NTs and the regeneration process could be monitored via MRI. (b) To identify our transplanted cells, BMSCs were genetically labeled with a green fluorescent protein (GFP). Scale bar: 100 μm. (c) The dramatically reduced red fluorescent signals of the ROS probe revealed that MnO_2_ NTs in the IHI nanoscaffold could effectively scavenge ROS in the defect area. Promoted cell proliferation was confirmed by the higher expression of proliferative marker Ki67 immunostaining. Scale bar: 50 μm. (d) The TGFβ–BMSC–IHI nanoscaffold could retain a significantly higher amount of cells after transplantation compared to other cell-transplantation groups by quantifying the number of remaining GFP^+^ cells in (c). (e) Histogram of the fluorescence intensity of ROS probe showed the effective consumption of ROS in the MnO_2_ NTs containing groups. (f) Quantification of Ki67^+^ cells in the defects. The quantifications in (e) and (f) were generated based on the fluorescence intensities in (c). All data are presented as mean ± SD (*n* = 5). ^*^P < 0.05, ^**^P < 0.01, ^***^P < 0.001.

With the promising results from *in vitro* chondrogenic assays, we next investigated the therapeutic potential of our IHI nanoscaffold for *in vivo* repair of injured cartilage with critically sized defects. Stem-cell therapies have been tested preclinically and clinically for treatment of cartilage injuries, but the low survival rate and insufficient chondrogenesis of stem cells *in vivo* remain as critical barriers [2,38,39]. In this regard, our 3D-IHI nanoscaffold represented a promising solution (Fig. 4a). Specifically, our MnO_2_ nanomaterial in the nanoscaffold initially enhanced stem-cell survival by scavenging ROS and then effectively promoted chondrogenesis at defect sites by incorporating deep drug delivery [37,40]. After establishing a standard rabbit cartilage-injury model, we injected IHI nanoscaffold into the injury sites and compared their therapeutic effects with control groups (Movie S3). To track transplanted cells *in vivo*, BMSCs were transfected with plasmids to express green fluorescence protein (GFP) before assembly into the nanoscaffold (Fig. 4b). At 7 days post-injury (DPI), we found significantly decreased ROS levels in our experiment (TGFβ–BMSC–IHI nanoscaffold) condition, specifically 6.51-fold and 5.40-fold lower signal intensities compared with the saline and BMSC spheroids injection only groups, respectively (Fig. 4c and e). Moreover, lower percentages of DNA damage and apoptosis were observed in the experimental group using the standard terminal deoxynucleotidyl transferase dUTP nick end labeling assay (Fig. S14). Furthermore, we assessed inflammation at the injury site, as previous reports have suggested a critical role for ROS in the activation of inflammatory signaling after cartilage injuries [36]. We verified an interesting anti-inflammatory effect *in vivo* from the BMSC spheroids (by comparing the BMSC spheroids and saline groups) and TGFβ–MnO_2_ NT (by comparing with the MnO_2_ and saline groups) based on the quantification of pro-inflammatory cytokines that included both interleukin-6 (IL-6) and tumor necrosis factor-α (TNF-α) (Fig. S15). Most importantly, because of the reduced ROS production, increased proliferative signaling and suppressed inflammation, the survival rate of the transplanted BMSCs was also improved in the 3D-IHI nanoscaffold group as quantified by the number of GFP-positive cells and higher expression of proliferative marker Ki67 around the injury sites (Fig. 4c, d and f). Therefore, we demonstrated a crucial step for stem-cell-based treatment of cartilage injuries *in vivo* by suppressing ROS and apoptotic signaling at injury sites.

**Figure 5.**
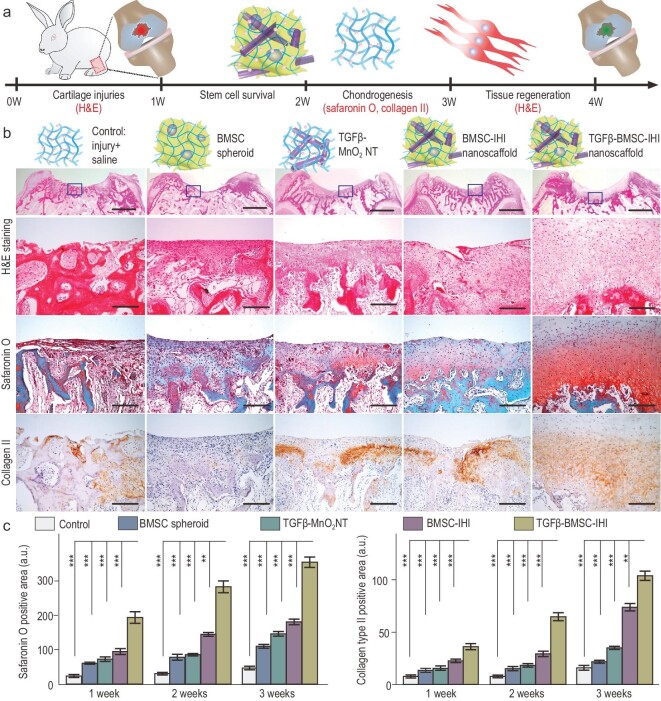
Enhancing *in vivo* chondrogenesis of BMSCs using 3D-IHI nanoscaffold. (a) Schematic illustration of the short-term chondrogenic differentiation after transplantation. (b) The *in vivo* chondrogenic differentiation was confirmed through hematoxylin and eosin (H&E), Safranin O staining, as well as Col II immunochemistry staining. Zoom-out scale bars: 2 mm, zoom-in scale bars: 200 μm. (c) Quantifications of cellular components (by Safranin O staining) and ECM components (by Col II immunostaining). These results collectively suggest that improved chondrogenic differentiation could be achieved through a MnO_2_ NT-templated cell assembly and homogeneous delivery of TGF-β3. All data are presented as mean ± SD (*n* = 5). ^**^P < 0.01, ^**^^*^P < 0.001.

We next investigated *in vivo* chondrogenesis of BMSCs transplanted using the 3D-IHI nanoscaffold-based approach *in vivo* (Fig. 5a). Our *in vitro* chondrogenic assay showed that incorporation of 3D cell–cell and cell–matrix interactions, as well as deep delivery of TGF-β3 in the 3D-IHI nanoscaffold, synergistically promoted the chondrogenesis of BMSCs. To verify the therapeutic effects, we included an additional experimental group treated with a TGFβ–BMSC–IHI nanoscaffold. The other four groups, saline (control), BMSC spheroids injection, TGFβ–MnO_2_ NT and BMSC–IHI nanoscaffold, were used as control groups. To examine chondrogenesis, hematoxylin and eosin (H&E), Safranin O staining, as well as Col II immunochemistry staining that selectively visualized chondrocytes were performed on all five animal groups at 7, 14 and 21 DPI, respectively (Figs 5b and S16a). Consistently with our *in vitro* cellular assays, we found a significantly higher population of chondrocytes at the injury sites of animals in the experimental group (TGFβ–BMSC–IHI nanoscaffold) compared with all four control groups by quantifications of Safranin O and Col II positive areas and cartilage thickness revealed by H&E staining (Figs 5c, S16b, S17 and S18). The extremely fast integration and regeneration indicated the significant advantages of our 3D-IHI nanoscaffold compared with the current tissue-engineering strategies.

Although our 3D-IHI nanoscaffold incorporates nanomaterials to enhance initial (>7 DPI) survival, integration and chondrogenic differentiation of stem cells, MnO_2_ NT in the nanoscaffold biodegraded timely within 30 DPI, thereby differentiating it from other scaffold-based cartilage-tissue-engineering approaches (Fig. S19a). The timely biodegradation of our nanomaterials in the 3D-IHI nanoscaffold could prevent immune reactions or mechanical mismatches in the long term [41]. In parallel, the degradation of MnO_2_ NT in our 3D-IHI nanoscaffold simultaneously released Mn^2+^ that facilitated MRI of the injured cartilage (Fig. S19b), which indicated that the release of TGF-β3 occurred for a relatively long period of time [42]. Therefore, our short-term stem-cell transplantation assay provided definitive proof of the dramatically accelerated regeneration of injured cartilages by the 3D-IHI nanoscaffold. Moreover, the nanomaterials in the 3D-IHI nanoscaffold were degraded in a timely manner to monitor the cartilage-repair process. Collectively, our results from the short-term *in vivo* cell-transplantation assays strongly suggested the crucial role of MnO_2_ NT in templating 3D-IHI nanoscaffold formation, remodeling of the hostile injury microenvironment and deep delivery of TGF-β3, thereby promoting the chondrogenic differentiation of the transplanted BMSCs.

**Figure 6.**
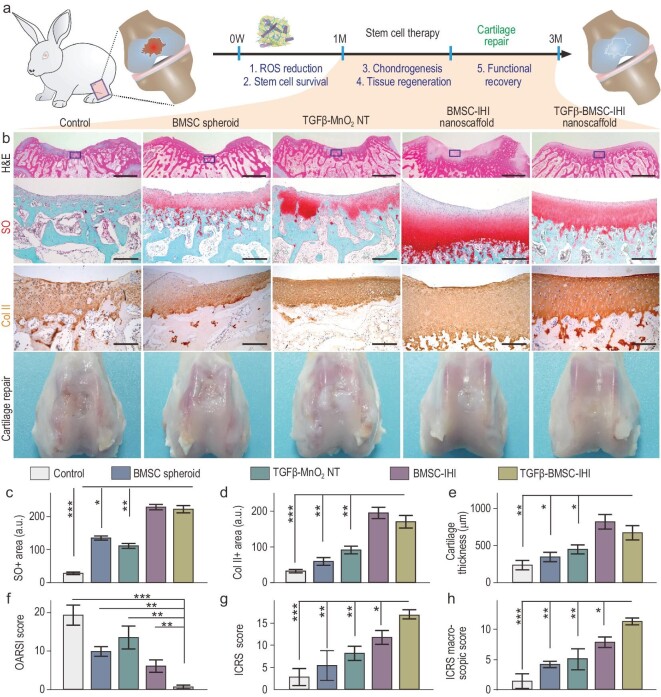
Accelerated cartilage repair by transplantation of 3D-IHI nanoscaffold. (a) A schematic diagram illustrating the long-term (3-month) cartilage-regeneration process. (b) The *in vivo* cartilage regeneration was characterized through H&E, Safranin O staining, Col II immunochemistry staining, as well as macroscopic views. Zoom-out scale bars: 2 mm, zoom-in scale bars: 200 μm. (c)–(h) Quantifications of cartilage thickness (by H&E staining) (c), cellular components (by Safranin O staining) (d) and ECM components (by Col II immunostaining) (e). Results of International Cartilage Repair Society (ICRS) macroscopic (f) and histologic scores (g) indicated significantly improved defect repair qualities in the TGFβ–BMSC–IHI nanoscaffold group. The reduced Osteoarthritis Research Society International (OARSI) scores revealed the TGFβ–BMSC–IHI nanoscaffold could prevent the deterioration of osteoarthritis (h). These results collectively suggest that improved cartilage regeneration could be achieved through a MnO_2_ NT-templated cell assembly and homogeneous delivery of TGF-β3. All data are presented as mean ± SD (*n* = 5). ^*^P < 0.05, ^**^P < 0.01, ^**^^*^P < 0.001.

To investigate long-term therapeutic effects, we performed three time-dependent [1, 2 and 3 months post-injury (MPI)] cell-transplantation assays using the same experimental (TGFβ–BMSC–IHI nanoscaffold) and control conditions (saline, BMSC spheroids, TGFβ–MnO_2_ NT and BMSC–IHI nanoscaffold) (Fig. 6a). Cartilage tissues harvested at the three time points (1, 2 and 3 MPI) were analysed for their structural integrity by a color photograph and H&E staining, cellular components by Safranin O staining and ECM compositions by immunostaining, all of which were crucial factors to assess cartilage regeneration (Fig. 6b). Strikingly, as early as 1 MPI, we observed smooth and near complete structural recovery of the injured cartilage tissue in the experimental group (TGFβ–BMSC–IHI nanoscaffold), which was not observed in any of the control groups (Fig. S20). This was consistent with a larger population of chondrocytes in H&E and Safranin O staining, as well as upregulated deposition of chondrogenic ECMs in Col II staining at injury sites in the experimental group animals across all three time points (Figs 6c–e and S21–S24). Additionally, significantly elevated International Cartilage Repair Society (ICRS) macroscopic and histologic scores in our experimental group compared with the control groups indicated that the MnO_2_ NT-templated assembly and homogeneous delivery of TGF-β3 had improved articular cartilage regeneration (Fig. 6f and g). Obvious joint degeneration was observed in the saline group. Thus, Osteoarthritis Research Society International (OARSI) scores were calculated for lesion assessments. Due to the promoted cartilage matrix production and reconstruction, the experimental group showed a dramatically reduced score, which revealed a better healing outcome of the critical-size defects at the early stage (1–3 months) and mitigated the deterioration of osteoarthritis (Fig. 6h). Considering the excellent therapeutic outcome of the 3D-IHI nanoscaffold, the desirable biocompatibility and the unique material properties provided by the MnO_2_ NTs, the MnO_2_ NT-templated cell-assembly method can be broadly applicable to treat other diseases and injuries (Figs S25 and S27).

## CONCLUSION

Cartilage injuries and diseases are often debilitating and currently lack effective treatments. To enhance stem-cell-based treatment of cartilage injuries and diseases, we developed a biodegradable nanomaterial-templated method to form a 3D-IHI nanoscaffold (Fig. S28). The incorporation of our biodegradable nanomaterial not only significantly accelerated the assembly of stem cells in 3D, but also integrated cell–cell and cell–matrix interactions, as well as a deep drug (TGF-β3) delivery function into the 3D-IHI nanoscaffold for effective modulation of BMSCs chondrogenesis *in vitro* and *in vivo*. Moreover, transplantation of the nanomaterial-templated 3D-IHI nanoscaffold promoted the repair of critically sized cartilage defects in a rabbit model at cellular and tissue levels. The excellent performance of the 3D-IHI nanoscaffold in controlling stem-cell fates *in vitro* and *in vivo* indicates the great potential of our therapeutic platform to accelerate the regeneration of cartilage and various other tissue injuries with low regenerative capacities.

## Supplementary Material

nwac037_Supplemental_Files
