# Supplementary material for: Injectable hybrid inorganic nanoscaffold as rapid stem cell assembly template for cartilage repair
Source: Natl Sci Rev. 2022 Feb 28;9(4):nwac037. doi: 10.1093/nsr/nwac037 (PMC8998491; doi:10.1093/nsr/nwac037)
Supplement: nwac037_Supplemental_Files [file nwac037_supplemental_files.zip › Supporting_Information.docx]

**Supporting Information**

**Injectable Hybrid Inorganic Nanoscaffold-templated Rapid Stem Cell Assembly for Cartilage Repair**

*Shenqiang Wang^1, 3, 4,†^, Letao Yang ^4, 6, †^, Bolei Cai^5, †^, Fuwei Liu^5^, Yannan Hou^4^, Hua Zheng^1^, Fang Cheng^1^, Hepeng Zhang^1,2,3^, Le Wang^5^, Xiaoyi Wang^5^, Qianxin Lv^5^, Liang Kong^5*^, Ki-Bum Lee^4*^  and Qiuyu Zhang^1,2,3*^*

^1^ Key Laboratory of Special Functional and Smart Polymer Materials of Ministry of Industry and Information Technology, School of Chemistry and Chemical Engineering, Northwestern Polytechnical University, Xi'an 710129, China.

Email: qyzhang@nwpu.edu.cn, Tel: +86-29-8843-1653

^2^ Research & Development Institute of Northwestern Polytechnical University in Shenzhen, Shenzhen 518057, China

^3^ Xi’an Key Laboratory of Functional Organic Porous Materials, School of Chemistry and Chemical Engineering, Northwestern Polytechnical University, Xi'an 710072, China

^4^ Department of Chemistry and Chemical Biology, Rutgers University, Piscataway, NJ 08854, USA

Email: kblee@rutgers.edu, Tel: +1-848-445-2081

^5^ State Key Laboratory of Military Stomatology & National Clinical Research Center for Oral Diseases & Shaanxi Key Laboratory of Oral Diseases, Department of Oral and Maxillofacial Surgery, School of Stomatology, The Fourth Military Medical University, Xi'an 710032, China

Email: liangkong2014@163.com, Tel: +86-029-84776019

^6^ Department of Biomedical Engineering, Columbia University, New York, NY 10032, USA

(^†^ These authors have contributed equally to this manuscript)

**KEYWORD**

Injectable nanoscaffold; 3D cell culture; Tissue engineering; Cartilage repair; Stem cell therapy;

**Table of Contents**

1. **Materials and Methods**
2. **Supplementary Figures, Table, and Videos**

**Figure S1. Characterizations of MnO_2_ nanotubes.**

**Figure S2. The cell assembly of 3D IHI nanoscaffold.**

**Figure S3. The morphorlogy of 3D****-IHI nanoscaffold.**

**Figure S4. 3D-IHI nanoscaffold enables both cell-cell interactions and cell-matrix interactions.**

**Figure S5. MnO_2_ nanotubes performed high TGF-β3 loading and stimulative release capacity.**

**Figure S6. 3D-IHI nanoscaffold formation in micro-well system.**

**Figure S7. Enhanced *in vitro* chondrogenic differentiation of BMSCs in the 3D-IHI nanoscaffold.**

**Figure S8. Alleviating oxidative stress by consuming ROS via the degradation of MnO_2_ nanotubes.**

**Figure S9. Cytotoxicity analysis of MnO_2_ nanotubes.**

**Figure S10. Blood compatibility of MnO_2_ nanotubes.**

**Figure S11. Morphological control of MnO_2_ nanostructures.**

**Figure S12. Pore structure control of MnO_2_ nanotubes.**

**Figure S13. A robust control over chondrogenesis.**

**Figure S14. MnO_2_ nanotubes protect cells from apoptosis *in vivo*.**

**Figure S15. Anti-inflammatory effect of the 3D-IHI nanoscaffold.**

**Figure S16. Enhancing *in vivo* chondrogenesis of BMSCs using 3D IHI nanoscaffold.**

**Figure S17. Enhanced short-term chondrogenic differentiation after transplantation.**

**Figure S18. Enhanced short-term chondrogenic differentiation via depositing ECM components after transplantation.**

**Figure S19. *In vivo* degradation and MRI-monitorable disease progression.**

**Figure S20. Long-term cartilage regeneration.**

**Figure S21. Enhancing *in vivo* cartilage regeneration using 3D-IHI nanoscaffold.**

**Figure S22. Enhanced long-term cartilage regeneration after transplantation.**

**Figure S23. Enhanced long-term cartilage regeneration via depositing ECM components after transplantation.**

**Figure S24. Reduced uncontrollable differentiation of BMSC *in vivo*.**

**Figure S25. *In vivo* compatibility assay of our 3D-IHI nanoscaffold.**

**Figure S26. Histomorphological evaluation of the main organs.**

**Figure S27. Histomorphological evaluation of the skin.**

**Figure 28. Summary of the development of 3D-IHI nanoscaffold-based treatment of cartilage injuries.**

**Supplementary Table 1.**

**Supplementary Table 2.**

**Materials and Methods**

**Materials.** All the chemicals, except additionally mentioned, were purchased from J & K Chemical. Gelatin (type A), 2', 7'-dichlorodihydrofluorescein diacetate (DCFH-DA), Live/Dead kit, and transforming growth factor-β3 (TGF-β3) were purchased from Sigma-Aldrich Co. (St Louis, MO, USA). PBS (pH 7.4), fetal bovine serum (FBS), Dulbecco’s modified Eagle medium (DMEM), Insulin-transferrin-selenium (ITS+) supplement, dexamethasone, and penicillin-streptomycin were purchased from Gibco Co., Ltd. (Carlsbad, CA, USA).

**BMSC culture.** Human Bone Marrow-derived Mesenchymal Stem Cell (BMSC) were expanded in a proliferation media containing high glucose DMEM (DMEM-HG) medium with 10% FBS, 100 IU mL^-1^ penicillin, and 100 µg mL^-1^ streptomycin at 37 °C in a 95% air, 5% CO_2_ atmosphere. To initiate the chondrogenic differentiation process, the media was replaced with differentiation media containing DMEM-HG medium with 10% FBS, 6.25 µg mL^-1^ Insulin-transferrin-selenium (ITS+) supplement, 100 nM dexamethasone, 100 IU ml^-1^ penicillin, and 100 µg ml^-1^ streptomycin [1]. Fresh media was exchanged every other day. BMSCs with passages 2-8 were used in the *in vitro* and *in vivo* studies.

**Preparation and characterization of MnO_2_ nanotubes.** MnO_2_ nanotubes were synthesized based on a previous protocol with minor modifications [2]. In a typical experiment, 2.5 mmol KMnO_4_ was added into 45 mL deionized water to form a homogeneous solution. Then, 0.5 mL concentrated HCl (37 wt.%) was added dropwise into the solution under magnetic stirring. The solution was transferred into a 100 mL Teflon-lined stainless steel autoclave and hydrothermally treated at 120 °C for 12 h. After the autoclave was cooled down to room temperature naturally, the samples were collected via centrifuge and washed several times with DI water. The obtained MnO_2_ nanotubes were dried via lyophilization. For the time-dependence study, the reaction was stopped at pre-determined time points and the corresponding brownish precipitates were obtained accordingly. MnO_2_ nanotube was diluted to 10 µg mL^-1^ for transmission electron microscopy (TEM, H-8000, Hitachi, Japan) assay. MnO_2_ nanotube was also imaged by field emission scanning electron microscopy (FE-SEM, SIGMA, Zeiss, Germany). The zeta potential of MnO_2_ nanotubes in an aqueous solution was measured by a dynamic light scattering instrument (Malvern Instruments, Malvern, UK). X-ray diffraction patterns were collected with a high-energy (115 keV, λ = 0.10798 Å) synchrotron X-ray beam.

**Measurement of TGF-*β*3 absorption by MnO_2_ nanotube.** We studied the TGF-*β*3 absorption capacity of MnO_2_ nanotubes with different inner channel diameters. 10 μL of MnO_2_ (MnO_2_-1, MnO_2_-2, MnO_2_-3, MnO_2_-4) nanotube aqueous solution (3 mg mL^-1^) was added into the solutions of transforming growth factor-*β*3 (TGF-*β*3, Sigma-Aldrich, stock concentration of 200 μg mL^-1^ in 0.5 mL PBS). The solutions turned brown immediately, and then they were continued to incubate at 37 °C for 12 h. To remove the MnO_2_ nanotube with absorbed TGF-*β*3, the solution was centrifuged for 3 times at 3000×g for 10 min, and the precipitates were removed each time until there were no visible precipitates anymore. 0.1 ml supernatant solution was transferred into a 96-well plate, and BCA (bicinchoninic acid assay, Thermo Fisher, A53226) was used to quantify the percentage of protein absorbed on nanotubes by a standard curve [3]. The assay was conducted strictly following the protocols from Thermo Fisher and the absorption at 570 nm was used to quantify the protein amount. The amount of TGF-*β*3 absorbed on MnO_2_ nanotube was calculated by subtracting the TGF-*β*3 concentration after MnO_2_ nanotube absorption from the original concentration. The percentage of TGF-*β*3 absorption was calculated by dividing the amount of TGF-*β*3 absorbed by the original TGF-*β*3 concentration. BCA protein assay was repeated 3 times.

**Controllable biodegradation of** **MnO_2_ nanotube.** To investigate the controllable biodegradability of MnO_2_ nanotube, we synthesized MnO_2_ nanotube with different degree of hollowness (MnO_2_-1, MnO_2_-2, MnO_2_-3, MnO_2_-4). Briefly, 10 μL of MnO_2_ nanotube aqueous solution (3 mg mL^-1^) was added into 3 mL of H_2_O_2_ (100 μM), and the UV-Vis spectrum of the solution was recorded at predetermined time intervals (3 h, 6 h, 12 h, 1d, 2d, 3d, 5d, and 7d). The percentage of nanotube remaining was determined by a standard curve of pure MnO_2_ nanotube solution.

To further mimic the transplantation conditions, the BMSCs were assembled with MnO_2_ nanotubes to investigate the redox-mediated biodegradation of MnO_2_ nanotubes without any exogenous stimuli factors. Briefly, 1 million BMSCs were suspended in 0.5 mL of MnO_2_ nanotubes (1mg mL^-1^ in DMEM media) solution and formed 3D IHI nanoscaffold using the micro-well system. A similar structure of carbon nanotube-cell nanoscaffold was formed using the same protocols as a control. Degradation kinetics of MnO_2_ nanotubes were investigated by monitoring color change of the 3D structure.

**Depletion of H_2_O_2_ catalyzed by MnO_2_ nanotubes.** The H_2_O_2_ scavenge effect of MnO_2_ nanotubes were conducted by mixing H_2_O_2_ (1 mM) and different concentration of MnO_2_ nanotubes (10, 20, 30, 50, 100 μg mL^-1^) solutions at 37 °C. At predetermined time intervals, the supernatant (50 µL) was collected and mixed with 100 µL Ti(SO_4_)_2_ solution [1.33 mL of 24 % Ti(SO_4_)_2_ and 8.33 mL of H_2_SO_4_ in 50 mL DI water] for 30 min. The concentration of H_2_O_2_ was obtained by measuring the absorbance at 405 nm and normalized by a standard curve of H_2_O_2_ solution.

**3D-IHI nanoscaffold fabrication in** **micro-well system.** The 3D-IHI nanoscaffold was generated using a microwell-facilitated spheroid formation followed by the fusion of the spheroids. Specifically, we first generated hydrogel microwell arrays by using a protocol previously described [4]. Briefly, Autocad-designed photomasks were printed on silicon wafers (Wanxiang SiliconPeak Electronics Co., China; SU-8 negative photoresist from MicroChem Corp., USA, was used). Next, the prepolymer solution of PDMS (10:1 ratio between the monomer and Sylgard 184 from Dow Corning Corp., USA, which is the curing agent) was transferred to the molds on a silicon wafer, with air bubbles removed using a vacuum for 3 times. Then PDMS was cured in an 85 °C oven for 3 hours. Next, hydrogel microwell arrays were fabricated by mixing a solution of PEG-DA (10% w/v, molecular weight of 700) and photoinitiator (1% w/v) onto a glass substrate [treated by 3-(trimethoxysilyl) propyl methacrylate]. The solution was pressed by the PDMS stamp under UV light exposure. To form the cell spheroids, BMSCs were seeded onto the hydrogel wells and incubated for 3 days at 37 °C before the free-floating cells were removed. In parallel, the 3D nano-hybrid stem cell spheroids were also formed in the micro-well plates. BMSCs were seeded onto the hydrogel wells with MnO_2_ nanotubes and incubated for 1 hour at 37 °C. The spheroids were then transferred onto a centrifuge tube to fuse into the injectable 3D-IHI nanoscaffold. To initiate the differentiation, the nanoscaffold was transferred to a fibronectin-coated glass substrate, allowed to adhere and proliferate for one day, followed by culture for two weeks under the differentiation media as described in the 2D differentiation study.

**Investigations of cell-cell and cell-matrix interactions.** BMSCs (1.0×10^6^) were seeded into a Petri dish as a negative control group. BMSCs with identical cell densities were seeded on the gelatin pre-coated glass. In parallel, BMSCs were also used to fabricate spheroids and BMSC-IHI nanoscaffold, respectively. The cell-cell and cell-matrix interactions were investigated by measuring the N-cadherin gene, Integrin β1, Wnt-related genes (GSK3β, β-catenin), FAK gene, and ERK1/2 gene via qRT-PCR.

**Intracellular ROS (H_2_O_2_) depletion.** The intracellular ROS scavenge ability of MnO_2_ nanotube was tested using BMSCs under oxidative microenvironment. Briefly, cells (5×10^4^) were incubated with different concentration of MnO_2_ nanotubes (10, 20, 30, 50, 100 μg mL^-1^) to form 3D-IHI nanoscaffold. Non-treatment group was chosen as a control. After incubation with H_2_O_2_ (100 μM) for 2 h, cells were then incubated with DCFH-DA (10 µM in FBS-free DMEM) for 20 min. Afterward, the intracellular ROS level was evaluated by detecting the fluorescence of DCF (λ_ex_=488 nm, λ_em_=525 nm) through a fluorescence microscope (IX53, Olympus, Japan) after rinsing with PBS for three times.

**Dye loading on MnO_2_ nanotube and MRI studies.** To study protein loading and release on MnO_2_ nanotube, rhodamine B-dextran was used as a model protein. Briefly, 0.2 mg rhodamine B-dextran (RhB-dextran) was added into 3.0 mL of MnO_2_ nanotube (1 mg mL^-1^) solution. After incubation at room temperature for 12 h, 5.0 mL PBS (Ph=7.4) was gradually added into the solution and RhB-dextran loaded MnO_2_ nanotube was centrifuged down at 3000×g for 5 min and extensively washed with PBS for 3 times to remove the residual RhB-dextran solution. Then the RhB-dextran-loaded MnO_2_ nanotube was re-suspended in 10 mL solution and re-assembled with gelatin (1 mg mL^-1^) at 37 ºC. To monitor the dye hold-up, RhB-dextran-loaded MnO_2_ nanotube was incubated with PBS for 12 h, then the fluorescence of the supernatant was detected by fluorescence spectra (Varian Cary Eclipse). The dye release was confirmed by degrading the RhB-dextran-loaded MnO_2_ nanotube using H_2_O_2_ (100 μM) PBS solution. The instant appearance of pink color from RhB-dextran proves the redox responsive release of RhB-dextran from MnO_2_ nanotube. RhB-dextran-loaded MnO_2_ nanotube before and after degradation was also spotted in a glass slide in a close-proximity and then imaged in the fluorescent microscope.

To investigate the dye distribution in the 3D-IHI nanoscaffold. 1 million BMSCs were suspended in 0.5 mL of RhB-dextran-loaded MnO_2_ nanotube (50 μg mL^-1^ in DMEM media) solution and formed 3D-IHI nanoscaffold using the micro-well system. In addition, BMSC spheroid was incubated in growth media containing 10 μg mL^-1^ RhB-dextran as control. After incubating for 1 h, the spheroids were washed with PBS and fixed with 4% (w/v) paraformaldehyde (PFA) for 24 h. Fixed spheroids were washed again and then cryopreserved in 30% (w/v) sucrose for 48 h. Whereafter, spheroids were embedded in paraffin and cut into 5μm-thick sections. The section slices were then imaged in the fluorescent microscope.

The magnetic resonance (MR) imaging was conducted on a 3T MRI scanner with the following parameters: field of view=5×3 cm^2^, matrix size=384×256, slice thickness=1 mm, echo time (TE)=8 ms, and repetition time (TR)=300 ms, 500 ms, 1000 ms, 2000 ms. The Mn concentration was determined by ICP-MS. To test the MR imaging performance, different concentrations of MnO_2_ nanotubes were incubated with H_2_O_2_ (500 μM) for 20 min, and the supernatant was collected and placed in Eppendorf tubes (2 mL volume) for MRI scanning. The *in vivo* MRI was conducted under anesthesia at different time points.

**Differentiation of BMSCs.** BMSCs (5×10^4^) were suspended with different concentration of gelatin coated MnO_2_ nanotubes (10, 20, 30, 50, 100 μg mL^-1^) in micro-well systems to form the 3D IHI nanoscaffold and incubated in both growth media and differentiation media for 14 days. To quantify the chondrogenesis markers (Sox9, Aggrecan, and Col-II), qRT-PCR was conducted by using GAPDH mRNA as a control.

***In vivo* transplantation of 3D-IHI nanoscaffold**. All animal work was conducted following the regulation of the Animal Care and Use Committee of The Fourth Military Medical University (approval number: SYXK 2020-004). Adult New Zealand white rabbits (3-3.5 kg, 3-month-old) were picked for the cartilage regeneration experiments. Animals are randomized without pre-knowledge of their behaviors and then assigned to different experimental groups without selection [5]. Then, a chondral defect model was created in 50 eligible rabbits for evaluating the survival, proliferation and differentiation, inflammatory responses, and regeneration effect of 3D-IHI nanoscaffold. After general anesthesia, a medial para-patellar incision was made to dislocate the patellar, and blunt dissection was performed to expose the articular surface, then chondral defects (6-mm diameter, 2-mm height) were created on the medial femoral condyle of the knees. Following induction of defect, saline control, BMSC spheroids, TGFβ-MnO_2_ NT, BMSC-IHI nanoscaffold, and TGFβ-BMSC-IHI nanoscaffold were individually injected into the wound site through an 18G syringe, and the synovial membrane and muscle around the surgical wound were sutured, respectively. After the operation, rabbits were allowed to move freely in their single cages and fed with standard food and water. At different time points (1, 2, 3, 4, 8, and 12 weeks later, rabbits were sacrificed for further study.

**qPCR Real-time quantitative RT-PCR:** Total RNA was extracted with TRIzol reagent (Invitrogen) and was reverse transcribed to cDNA with Superscript III Reverse Transcriptase (Invitrogen). Conventional quantitative RT-PCR was performed using an SYBR Green PCR Master Mix (Applied Biosystems) on a StepOnePlus Real-time PCR System (Applied Biosystems), and the resulting Ct values were normalized to GAPDH. Standard cycling conditions were used for all reactions with a melting temperature of 60°C. The primer sequence for the genes which were analyzed are included in the Supplemental Information (Table S1).[6] (Error bars represent mean ±s.d.; n=4)

**Statistical analysis.** All the experimental data were statistically analyzed and the results were expressed as a mean ± standard deviation (SD). Statistical differences were determined using one-way ANOVA followed by a Bonferroni post hoc test for multiple comparisons with SPSS, version 24 (IBM). Data were considered as statistically significant difference when **p* < 0.05, ***p* < 0.01, and ****p*<0.001 versus the indicated group.

**References**

1. Xu J, Li J, Lin S*, et al.* Nanocarrier‐mediated codelivery of small molecular drugs and siRNA to enhance chondrogenic differentiation and suppress hypertrophy of human mesenchymal stem cells. *Adv Funct Mater* 2016; **26**: 2463-72.

2. Truong TT, Liu Y, Ren Y*, et al.* Morphological and crystalline evolution of nanostructured MnO_2_ and its application in lithium–air batteries. *ACS Nano* 2012; **6**: 8067-77.

3. Drinnan CT, Zhang G, Alexander MA*, et al.* Multimodal release of transforming growth factor-β1 and the BB isoform of platelet derived growth factor from PEGylated fibrin gels. *J Control Release* 2010; **147**: 180-6.

4. Lee JM, Yang L, Kim E-J*, et al.* Generation of uniform-sized multicellular tumor spheroids using hydrogel microwells for advanced drug screening. *Sci Rep* 2018; **8**: 1-10.

5. Shi W, Sun M, Hu X*, et al.* Structurally and functionally optimized silk‐fibroin–gelatin scaffold using 3D printing to repair cartilage injury in vitro and in vivo. *Adv Mater* 2017; **29**: 1701089.

6. Yang L, Conley BM, Cerqueira SR*, et al.* Effective Modulation of CNS Inhibitory Microenvironment using Bioinspired Hybrid‐Nanoscaffold‐Based Therapeutic Interventions. *Adv Mater* 2020; **32**: 2002578.

**Supplementary Figures**


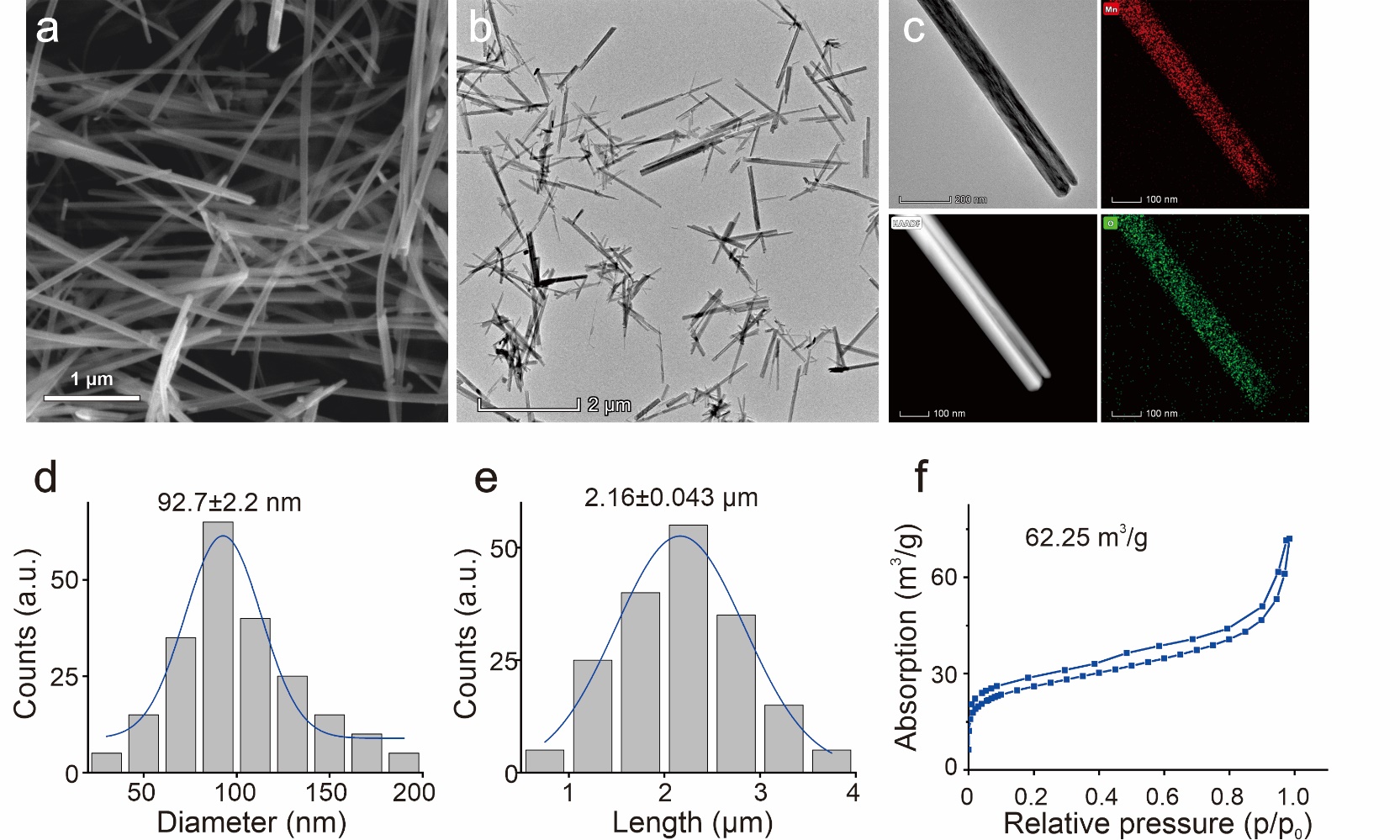


**Figure S1. Characterizations of MnO_2_ nanotubes.** a) The representative scanning electron microscopy (SEM) image of MnO_2_ nanotubes. b) The representative Transmission Electron Microscope (TEM) image of MnO_2_ nanotubes. c) The zoom-in TEM image and Energy Dispersive Spectroscopy (EDS) elemental mapping of MnO_2_ nanotubes. d) The diameter of MnO_2_ nanotubes. e) The length of MnO_2_ nanotubes. f) The high surface area of MnO_2_ nanotubes was confirmed via BET analysis.


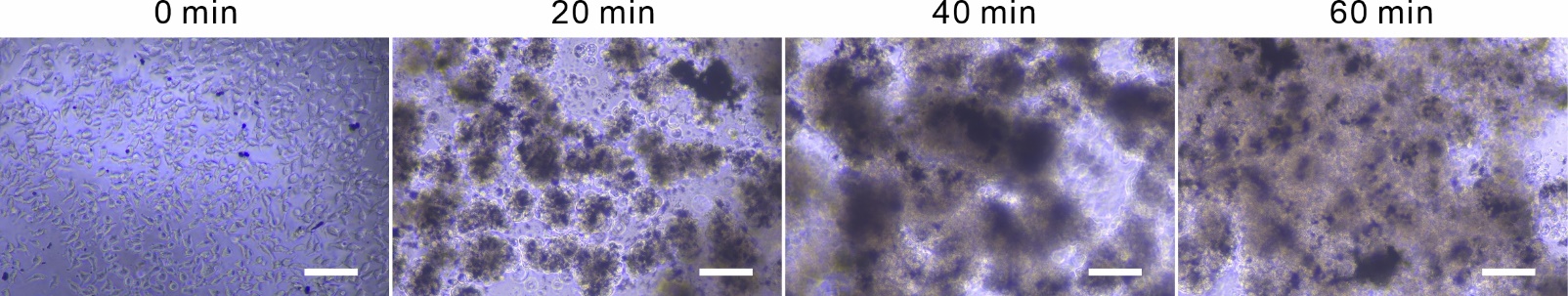


**Figure S2. The cell assembly of 3D IHI nanoscaffold.** The MnO_2_ nanotube templated cell assembly process was monitored via a microscope. Scale bar: 50 μm.


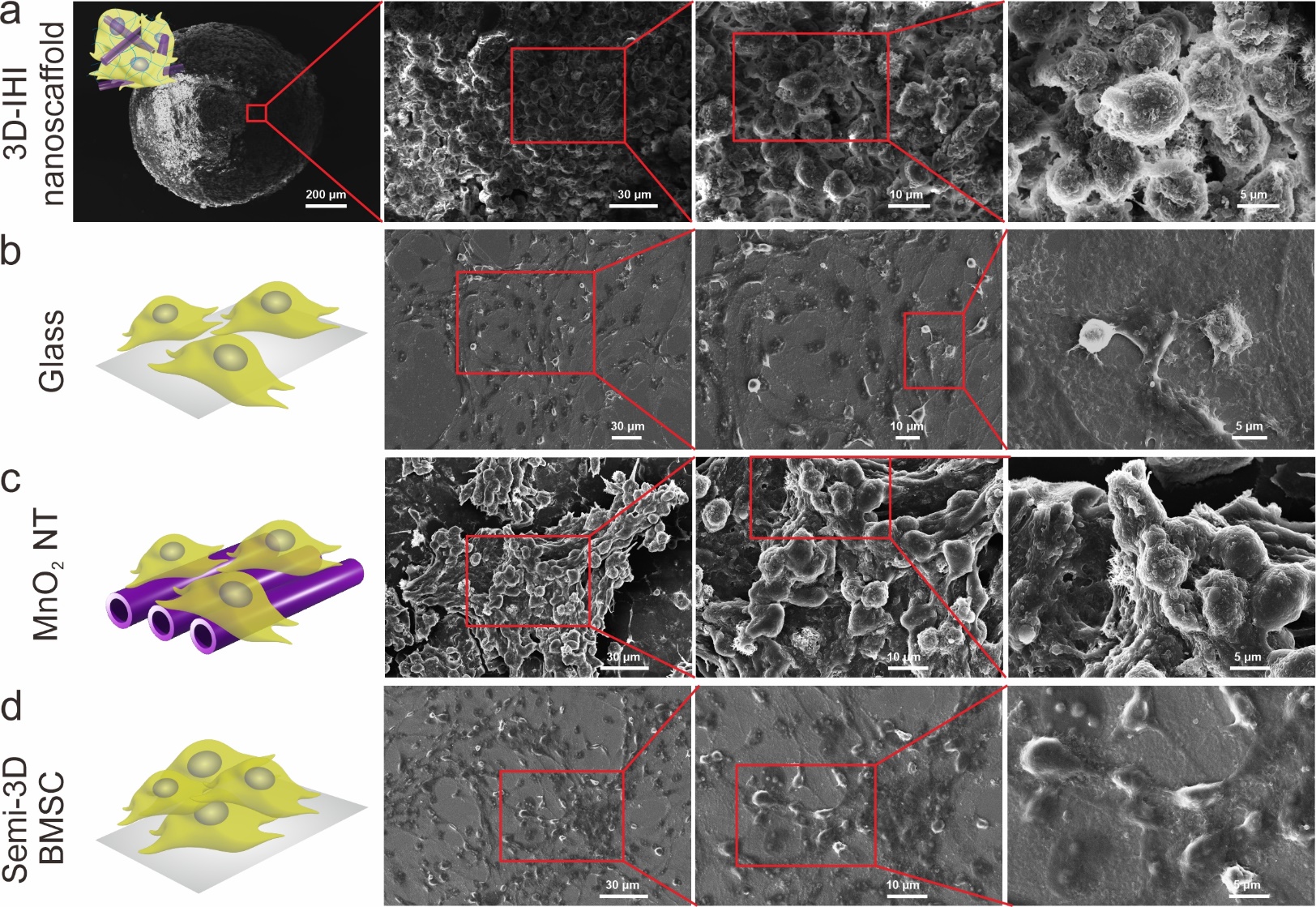


**Figure S3.** **The morphology of 3D-IHI nanoscaffold.** a-d) Field-emission scanning electron microscopy (FESEM) images of 3D-IHI nanoscaffold (a), BMSCs incubated in the gelatin-formulated media conditions (Glass) (b), MnO_2_ NT (c), and the assembled cells after prolonged incubation of BMSCs in media-alone conditions (d).


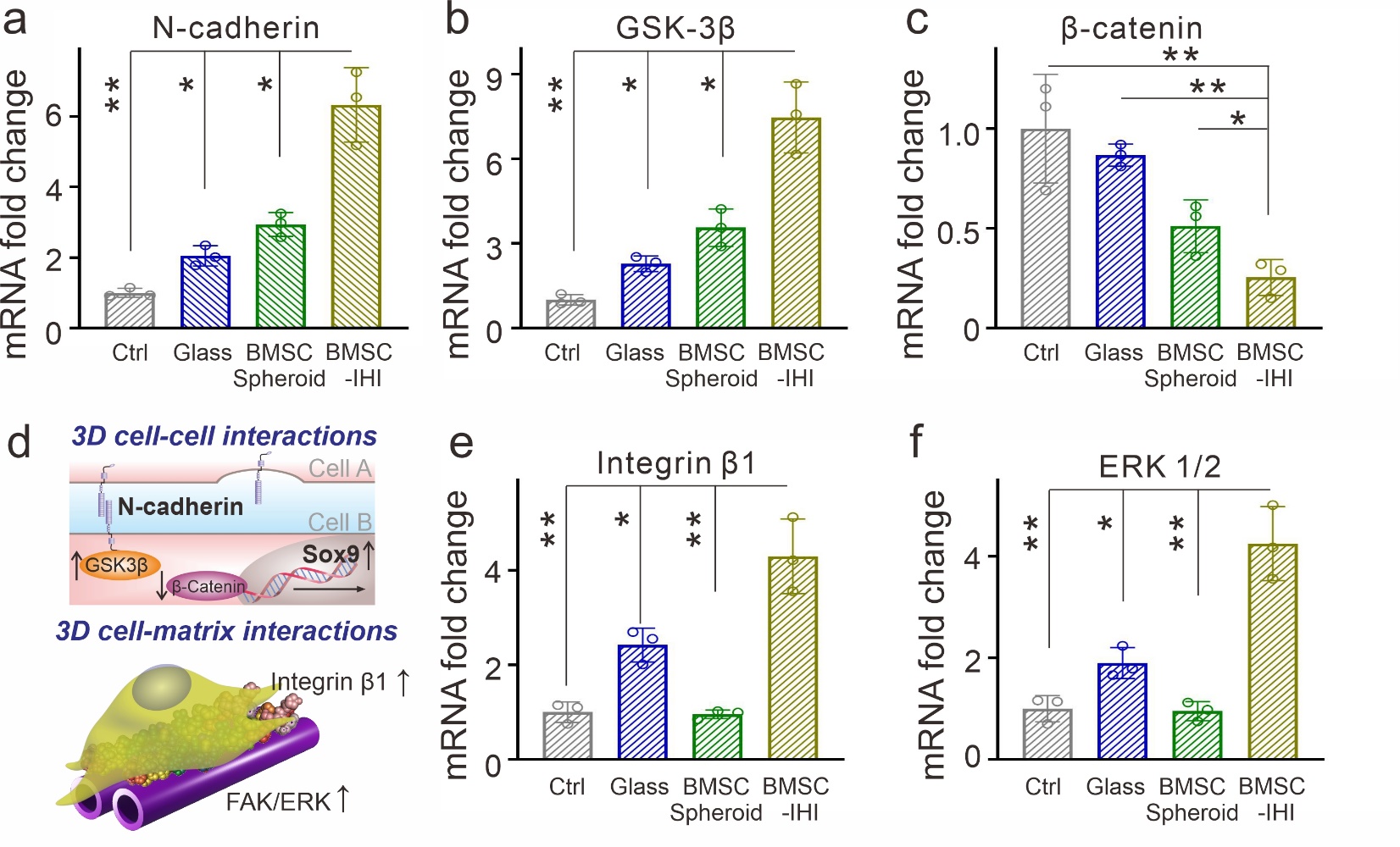


**Figure S4. 3D-HI nanoscaffold enables both cell-cell interactions and cell-matrix interactions.** a-c) The cell-cell interactions in 3D-IHI nanoscaffold the expression of N-cadherin, which is believed to enhance chondrogenesis through Wnt signaling pathways (a). The up-regulated expression of Wnt inhibitory gene (glycogen synthase kinase-3β, GSK3β) (b) and down-regulation of Wnt-related gene (β-catenin) (c) were confirmed via qRT-PCR measurement. d) Schematic diagram indicating the cell-cell and cell-matrix interactions in the 3D-IHI nanoscaffold. e-f) The cell-matrix interactions in the 3D-IHI nanoscaffold up-regulate the expression of Integrin β1 (e) and ERK1/2 (f). All data are presented as mean ± SD (n=4). *p < 0.05, **p < 0.01, ***p < 0.001.

**
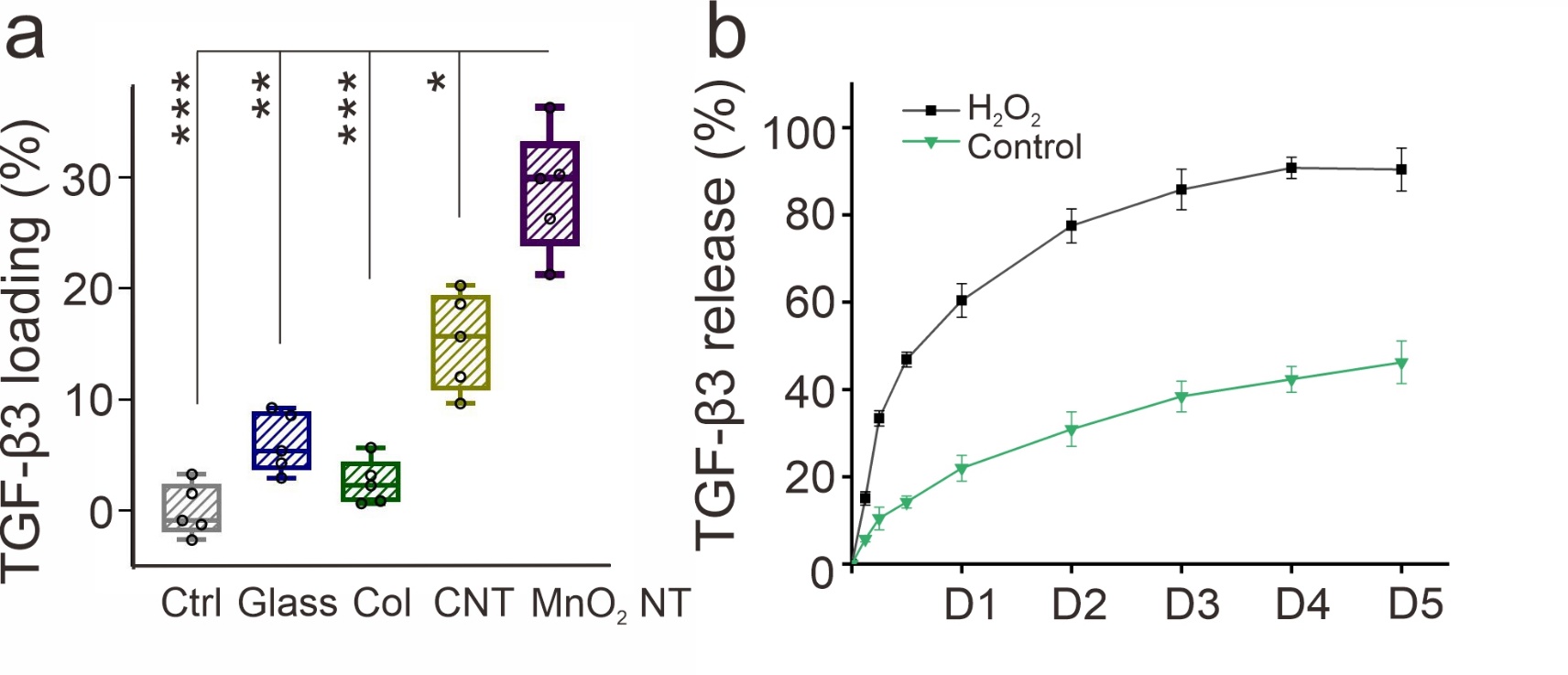
**

**Figure S5. MnO_2_ nanotubes performed high TGF-β3 loading and stimulative release capacity.** a) Bicinchoninic acid assay indicates the enhanced absorption toward TGF-β3 from MnO_2_ nanotube compared to control groups [glass, collagen fibers (Col) and carbon nanotube (CNT)]. b) The release of TGF-β3 could be significantly accelerated in the oxidative microenvironment (100 μM H_2_O_2_). All data are presented as mean ± SD (n=3). *p < 0.05, **p < 0.01, ***p < 0.001.


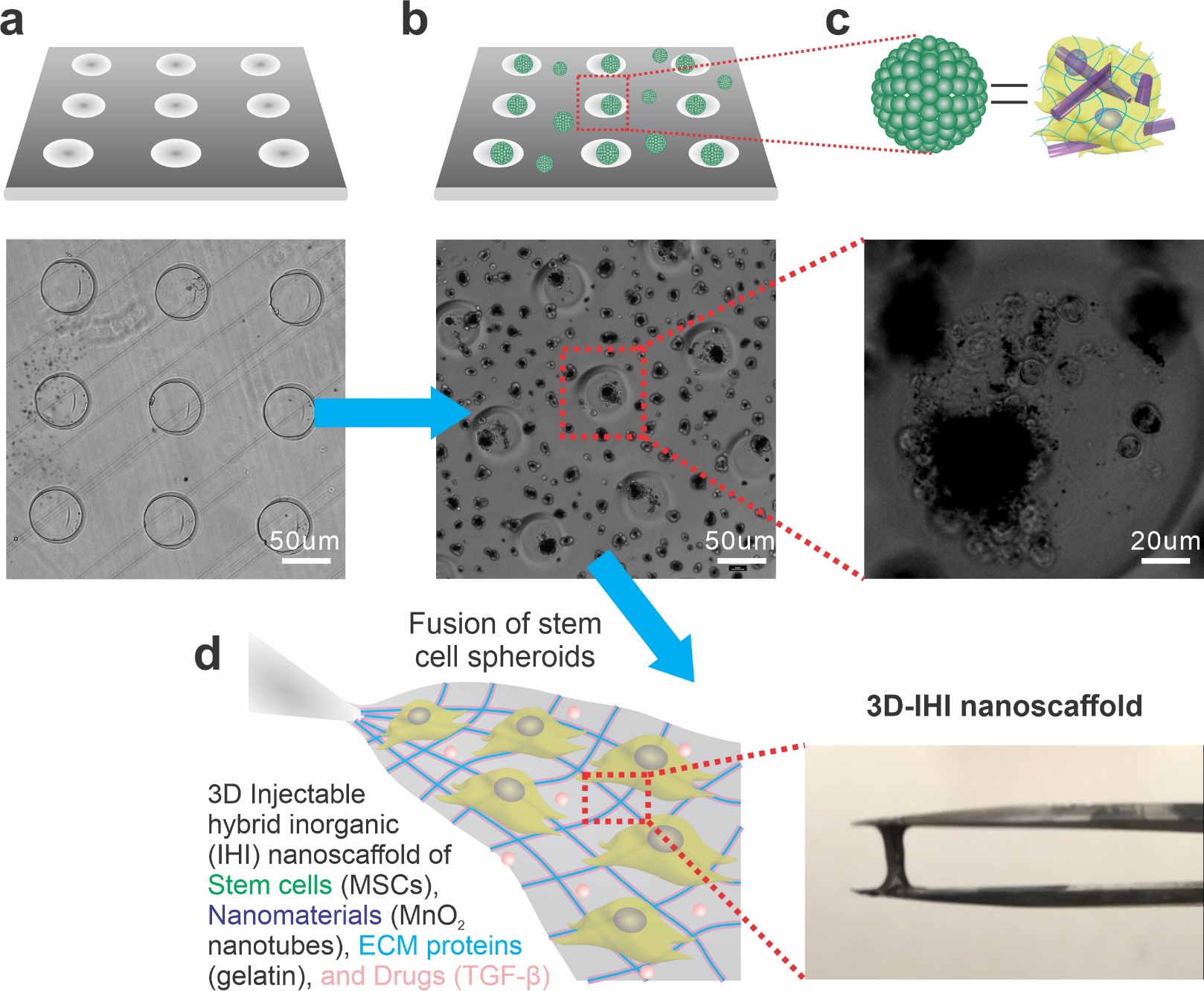


**Figure S6. 3D-IHI nanoscaffold formation in micro-well system.** a) The schematic illustration and image of the micro-well. b) The formation of 3D nano-hybrid stem cell spheroids in the micro-well plates. c) Zoom-in image of the formed 3D nano-hybrid stem cell spheroids. d) The 3D-IHI nanoscaffold was generated using a microwell-facilitated spheroid formation followed by the fusion of the spheroids.


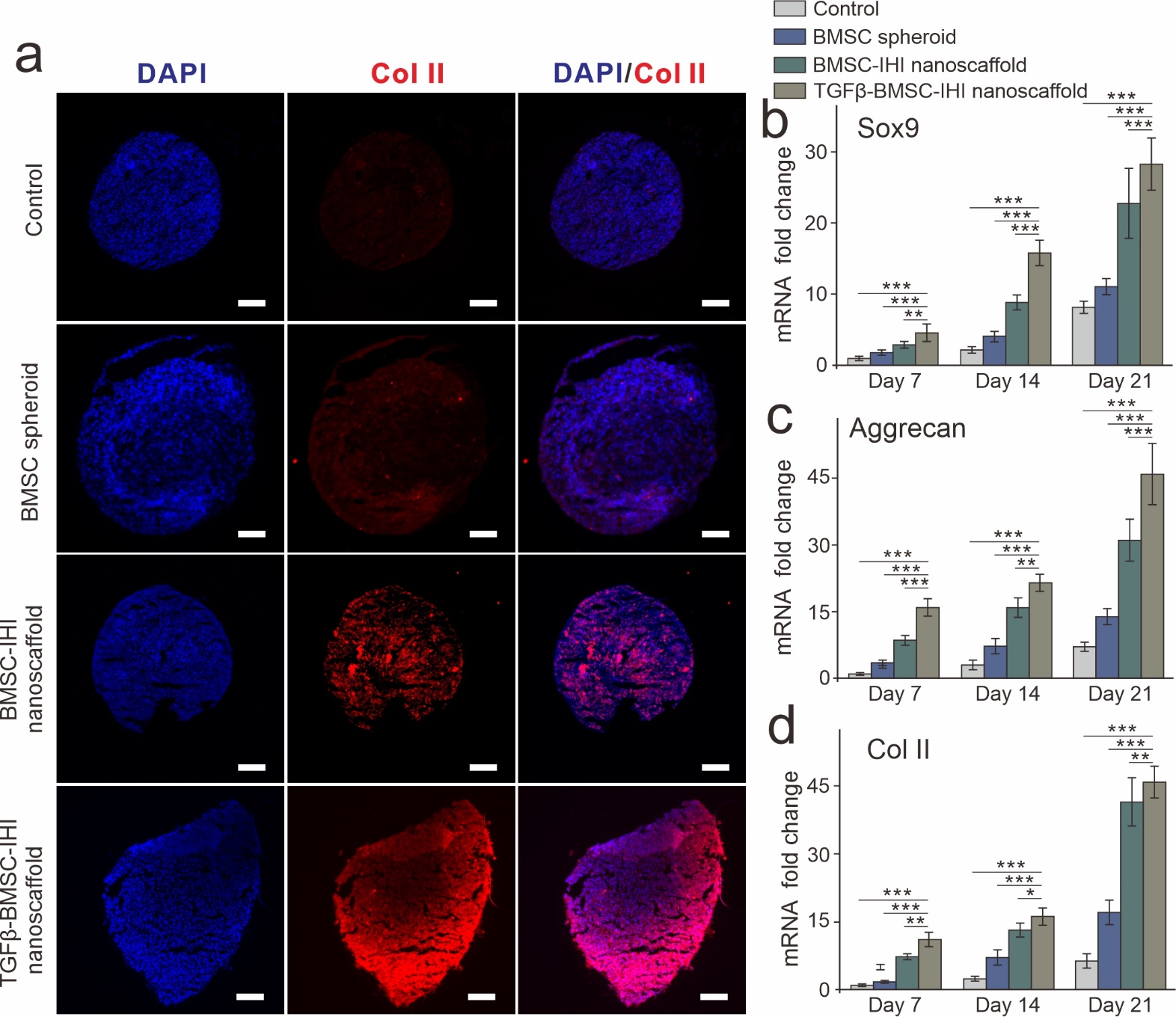


**Figure S7. Enhanced *in vitro* chondrogenic differentiation of BMSCs in the 3D-IHI nanoscaffold.** a) Immunostaining results on chondrogenic markers (Col II, labeled with red) on day 14 demonstrate significant enhancement of chondrogenesis of BMSC differentiated in the TGFβ-BMSC-IHI nanoscaffold compared to the control groups. Scale bar: 200 μm. b-c) The expression of chondrogenic genes including Sox9 (b), Aggrecan (c), and Col II (d) were characterized via qRT-PCR measurement throughout the 21 days *in vitro* differentiation study. All data are presented as mean ± SD (n=4). *p < 0.05, **p < 0.01, ***p < 0.001.

**
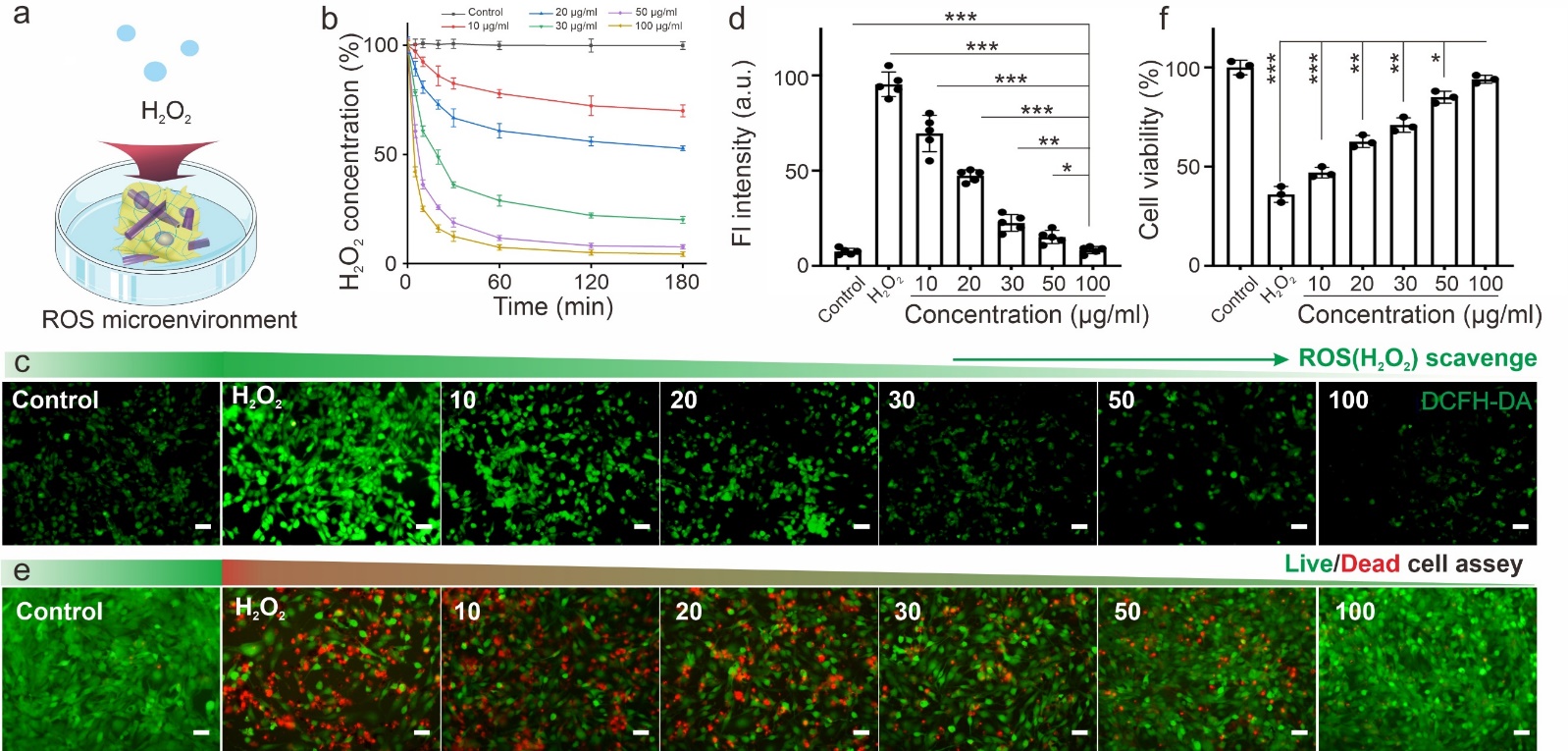
**

**Figure S8. Alleviating oxidative stress by consuming ROS via the degradation of MnO_2_ nanotubes.** a) Schematic illustration showing that the 3D-IHI nanoscaffold was exposed to the ROS microenvironment simulating medium containing 100 µM H_2_O_2_. b) Quantitative analysis of the residual H_2_O_2_ in the solution after applying different concentration of MnO_2_ nanotubes, which was characterized by a colorimetric measurement. c-d) The intracellular ROS depletion assay (c) and quantitative analysis (d) were monitored via a ROS probe (DCFH-DA) after incorporating varied concentrations of MnO_2_ nanotubes, validating the rapid consumption of H_2_O_2_. e-f) The protective function of MnO_2_ nanotubes to BMSCs against the ROS microenvironment was conducted via live-dead staining (e) and cell apoptotic assay (f). Scale bar: 50 μm. All data are presented as mean ± SD (n=5). *p < 0.05, **p < 0.01, ***p < 0.001.


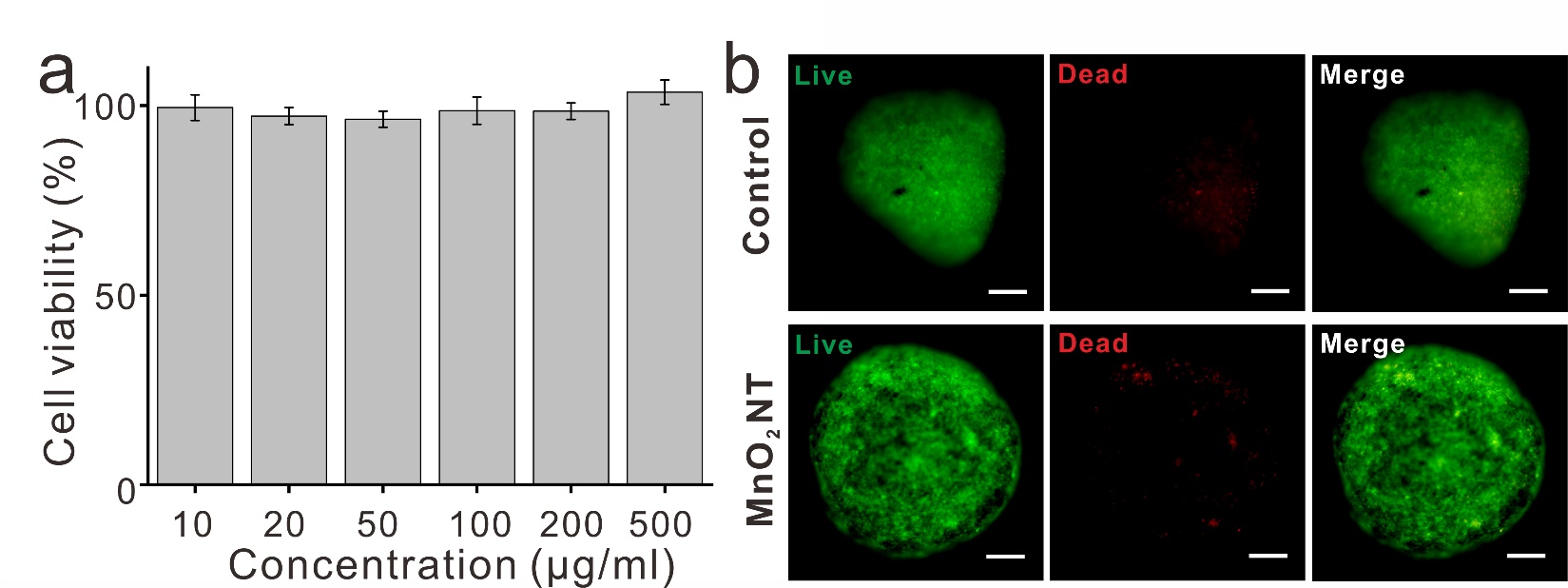


**Figure S9.** **Cytotoxicity analysis of MnO_2_ nanotubes.** a-b) The cell viability of BMSCs incubated with MnO_2_ nanotubes for 48 h based on cell apoptotic assay (a) and live-dead staining (b). Scale bar: 100 μm. All data are presented as mean ± SD (n=3).


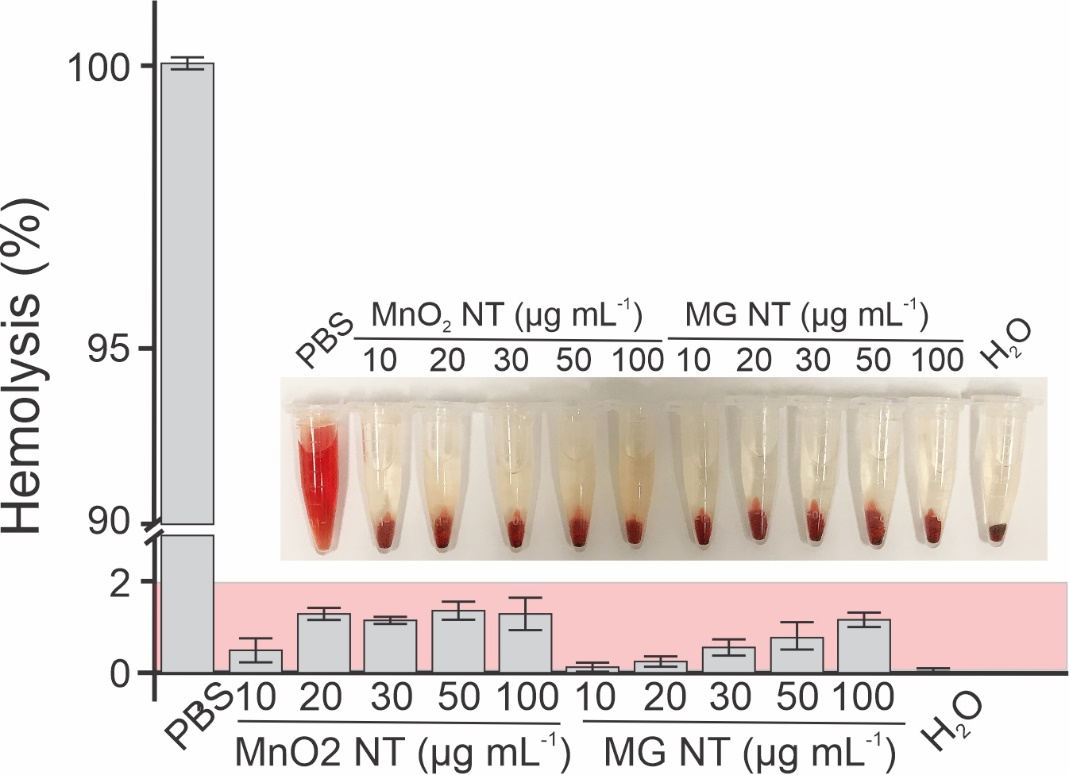


**Figure S10. Blood compatibility of MnO_2_ nanotubes.** Hemolytic ratio of MnO_2_ nanotubes and gelatin coated MnO_2_ (MG) nanotubes with different concentrations (10, 20, 30, 50, 100, 200 µg ml^-1^). Inset: digital photograph of hemolytic test. All data are presented as mean ± SD (n=3).


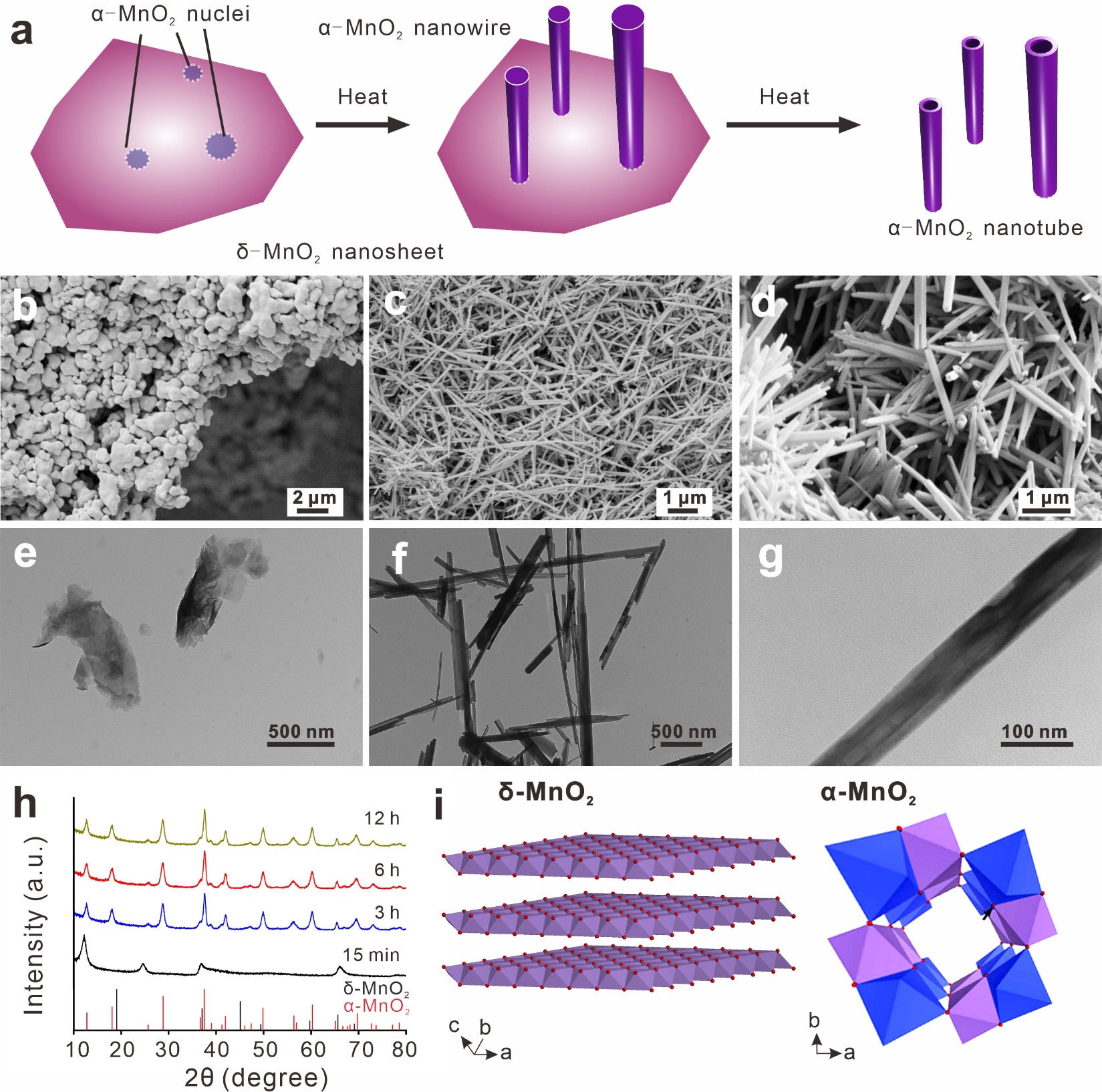


**Figure S11. Morphological control of MnO_2_ nanostructures.** a) Schematic diagram showing the morphology and phase transition of MnO_2_ nanostructures. b-d) Representative SEM images of MnO_2_ nanosheets (b), nanowires (c), and nanotubes (d). e-g) Representative TEM images of MnO_2_ nanosheets (e), nanowires (f), and nanotubes (g). h) XRD patterns of the MnO_2_ products at different time points. The sticks highlight the peak positions and relative peak intensities of the standard powder XRD patterns of α-MnO_2_ (red) and δ-MnO_2_ (black) for reference. i) Schematic illustrations of crystal structures of δ-MnO_2_ and α-MnO_2_.


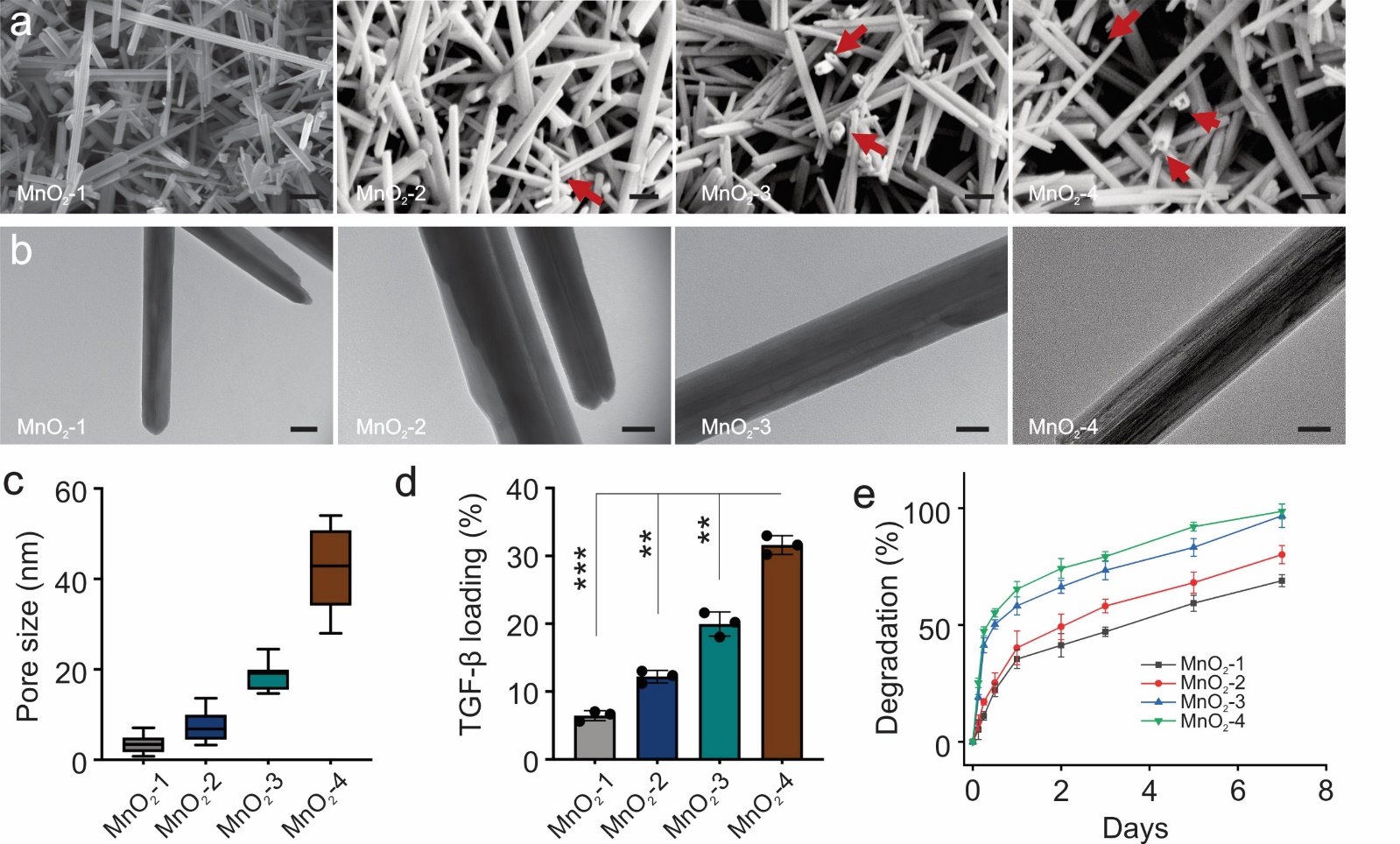


**Figure S12. Pore structure control of MnO_2_ nanotubes.** a) Representative SEM images of MnO_2_ nanotubes with different pore structure by controlling the addition of HCl and reaction time. Scale bar: 100 nm. b) Representative TEM images of MnO_2_ nanotubes with different pore structure. Scale bar: 20 nm. c) The pore size variations of MnO_2_ nanotubes, which were counted from SEM images. d) Larger pore enhances the loading capacity of TGF-β3. e) Tunable degradation kinetics were controlled by modulating the porosity of MnO_2_ nanotubes in the oxidative media (100 µM H_2_O_2_). All data are presented as mean ± SD (n=3). **p < 0.01, ***p < 0.001.


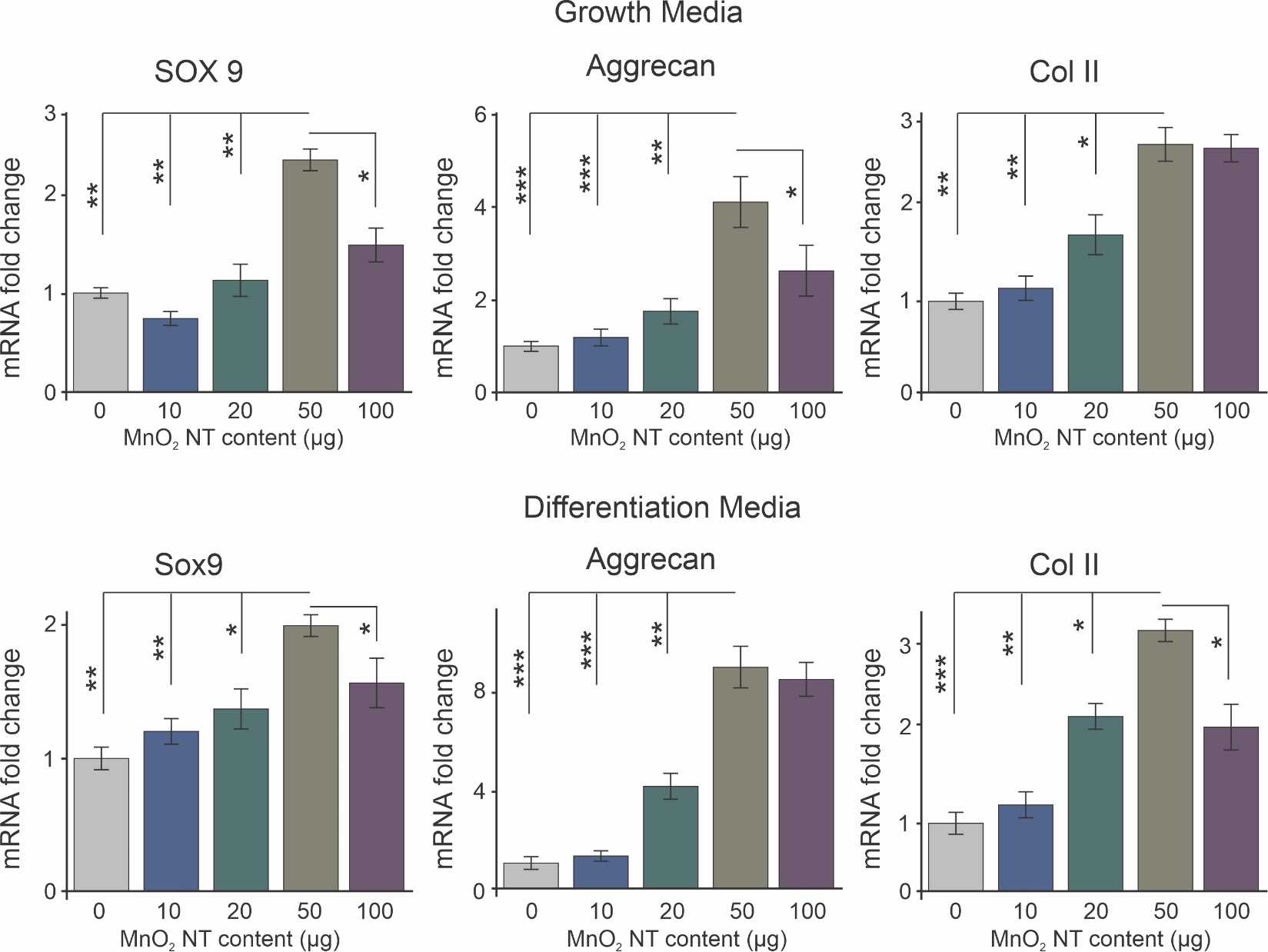


**Figure S13. A robust control over chondrogenesis.** The expression of chondrogenic genes including SOX 9, Aggrecan, and Col II were characterized via qRT-PCR measurement. The BMSCs were incubated with different contents of MnO_2_ nanotubes in both growth media and differentiation media. All data are presented as mean ± SD (n=4). *p < 0.05, **p < 0.01, ***p < 0.001.


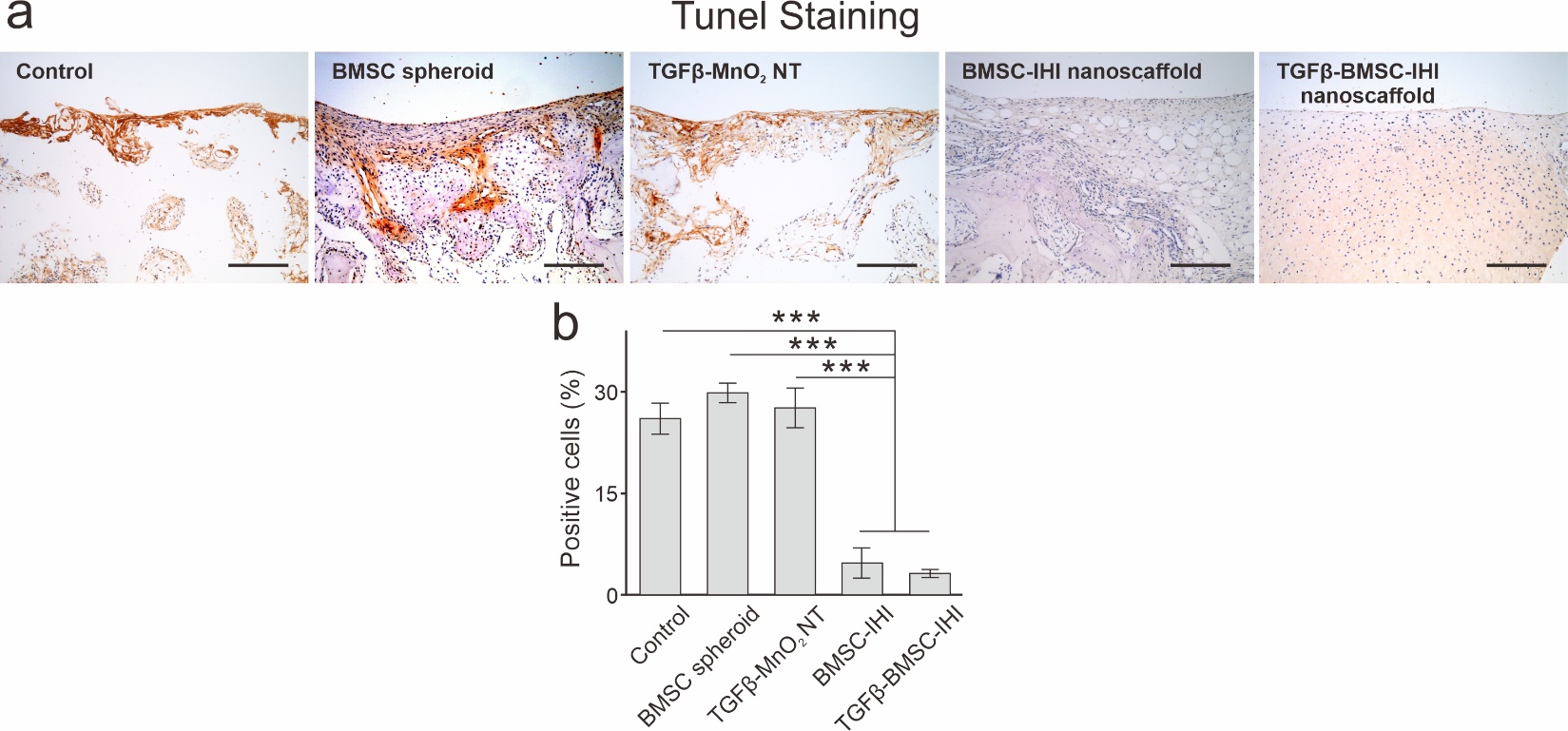


**Figure S14. MnO_2_ nanotubes protect cells from apoptosis *in vivo*.** a-b) Tunel staining images (a) and quantitative analysis of positive cells (b) around the injury sites. As a result of ROS reduction, the apoptotic signalings at the injury site were lowered in our BMSC-IHI nanoscaffold and TGFβ-BMSC-IHI nanoscaffold groups compared to the control groups (saline, BMSC spheroid, TGFβ-MnO_2_ NT). All data are presented as mean ± SD (n=5). ***p < 0.001.


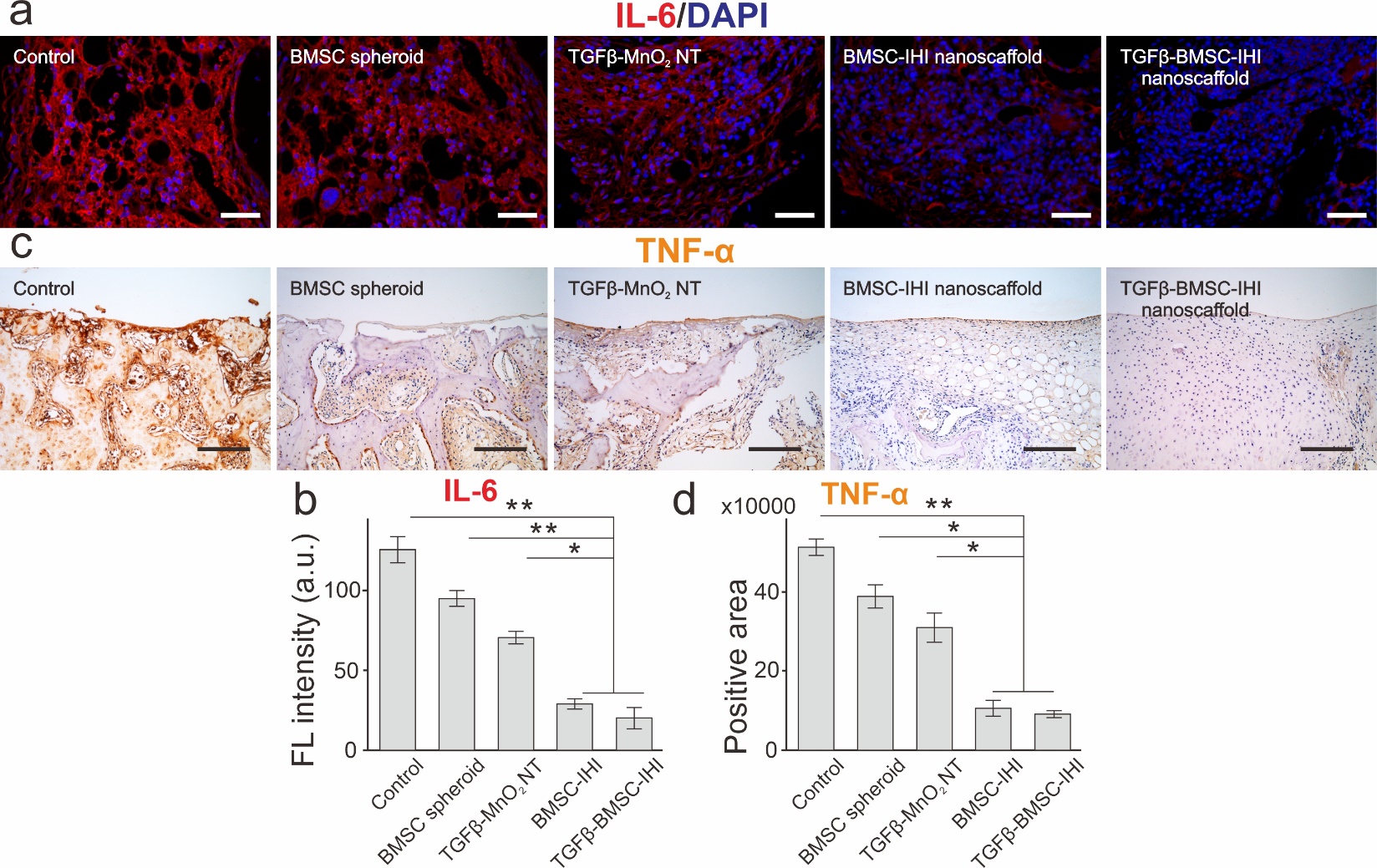


**Figure S15. Anti-inflammatory effect of the IHI-nanoscaffold.** a-b) IL-6 immunostaining images (a) and quantitative analysis (b) in the cartilage tissue slices. c-d) TNF-α immunostaining images (c) and quantitative analysis (d) in the cartilage tissue slices. Scale bar: 100 μm. The anti-inflammatory effect of BMSC and MnO_2_ nanotubes were effectively combined and demonstrated a further enhanced reduction of inflammatory cytokines at the injury site in the 3D-IHI nanoscaffold group. Scale bar: 200 μm. All data are presented as mean ± SD (n=5). *p < 0.05, **p < 0.01.


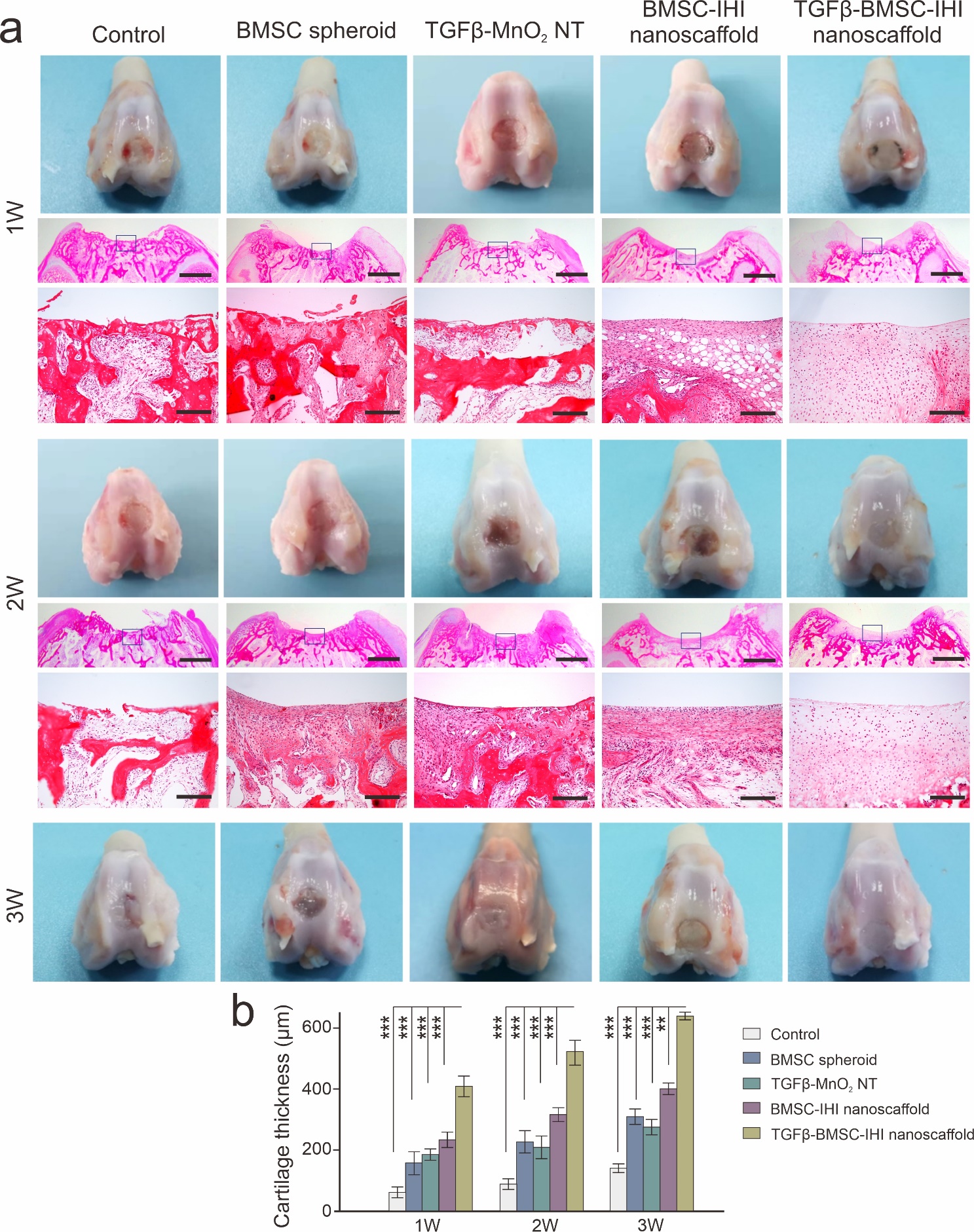


**Figure S16. Enhancing *in vivo* chondrogenesis of BMSCs using 3D-IHI nanoscaffold.** a) The regenerated cartilage tissues were characterized by Hematoxylin and Eosin (H&E) and macroscopic images. Zoom out scale bar: 2 mm, Zoom in scale bar: 200 μm. b) Quantifications of cartilage thickness from H&E staining. All data are presented as mean ± SD (n=5). **p < 0.01, ***p < 0.001.


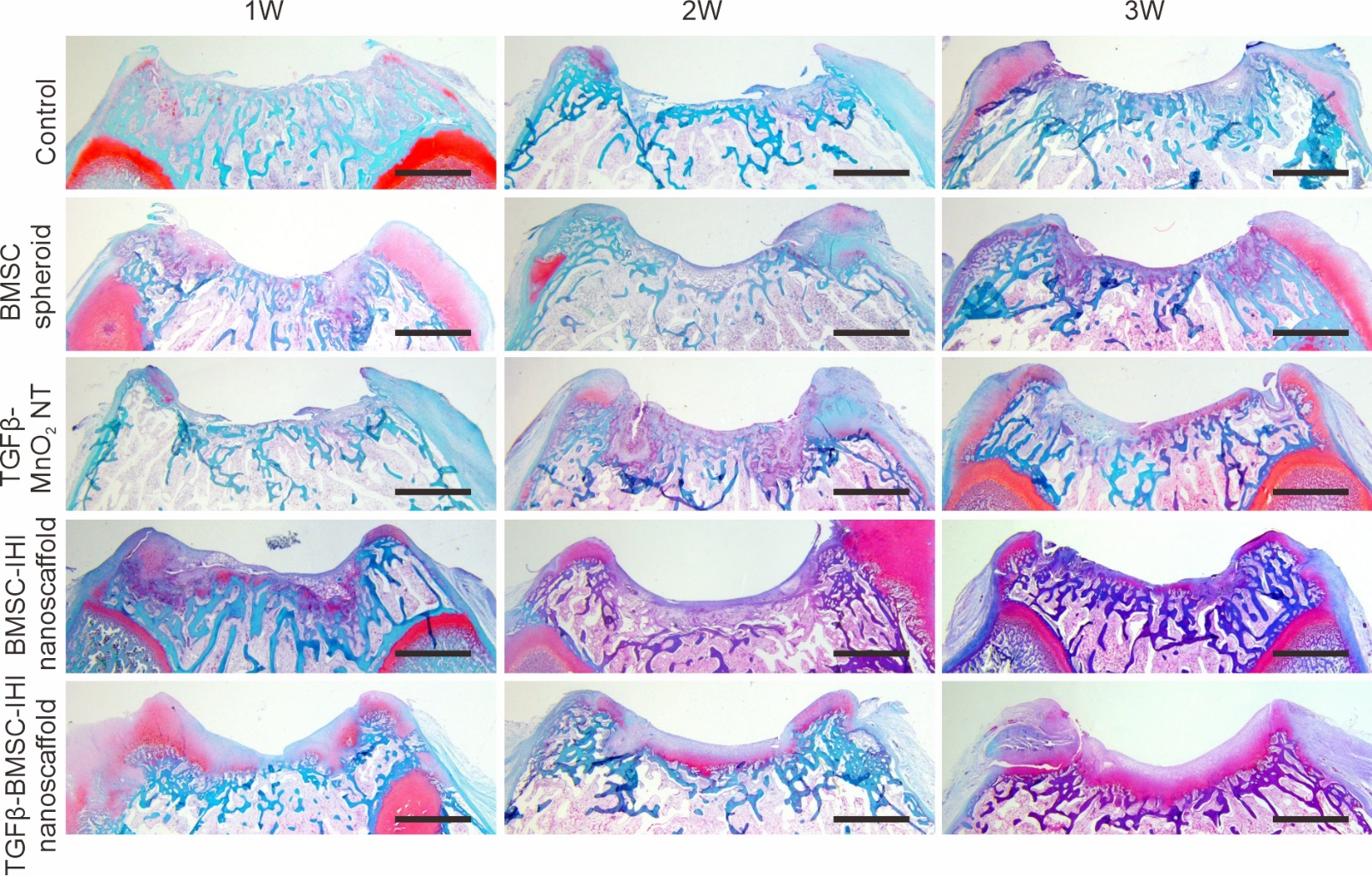


**Figure S17. Enhanced short-term chondrogenic differentiation after transplantation.** The *in vivo* chondrogenic differentiation was characterized through Safranin O staining. Scale bar: 2 mm.


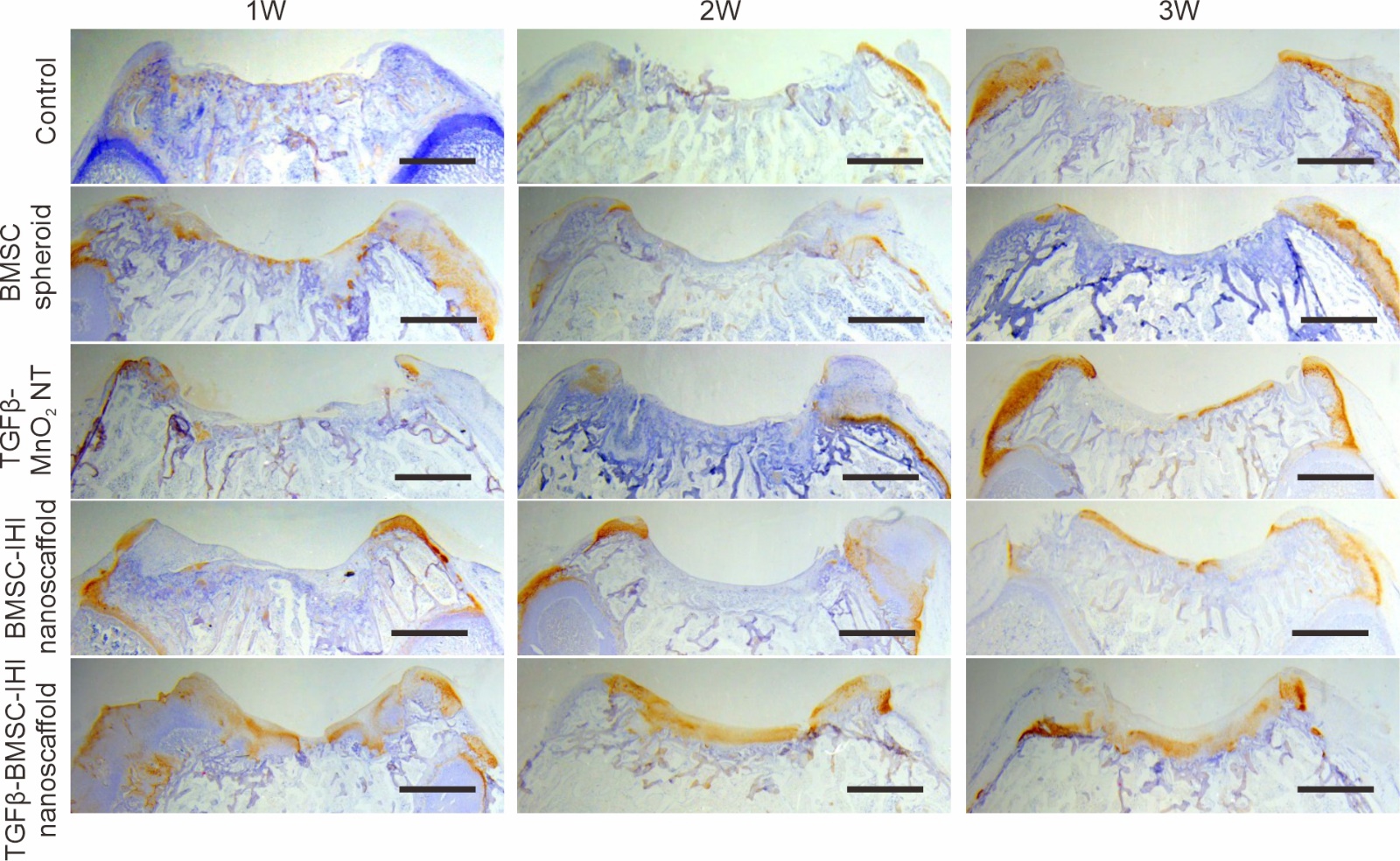


**Figure S18. Enhanced short-term chondrogenic differentiation via depositing ECM components after transplantation.** The *in vivo* chondrogenic differentiation was characterized through Collagen-II (Col II) immunochemistry staining. Scale bar: 2 mm.


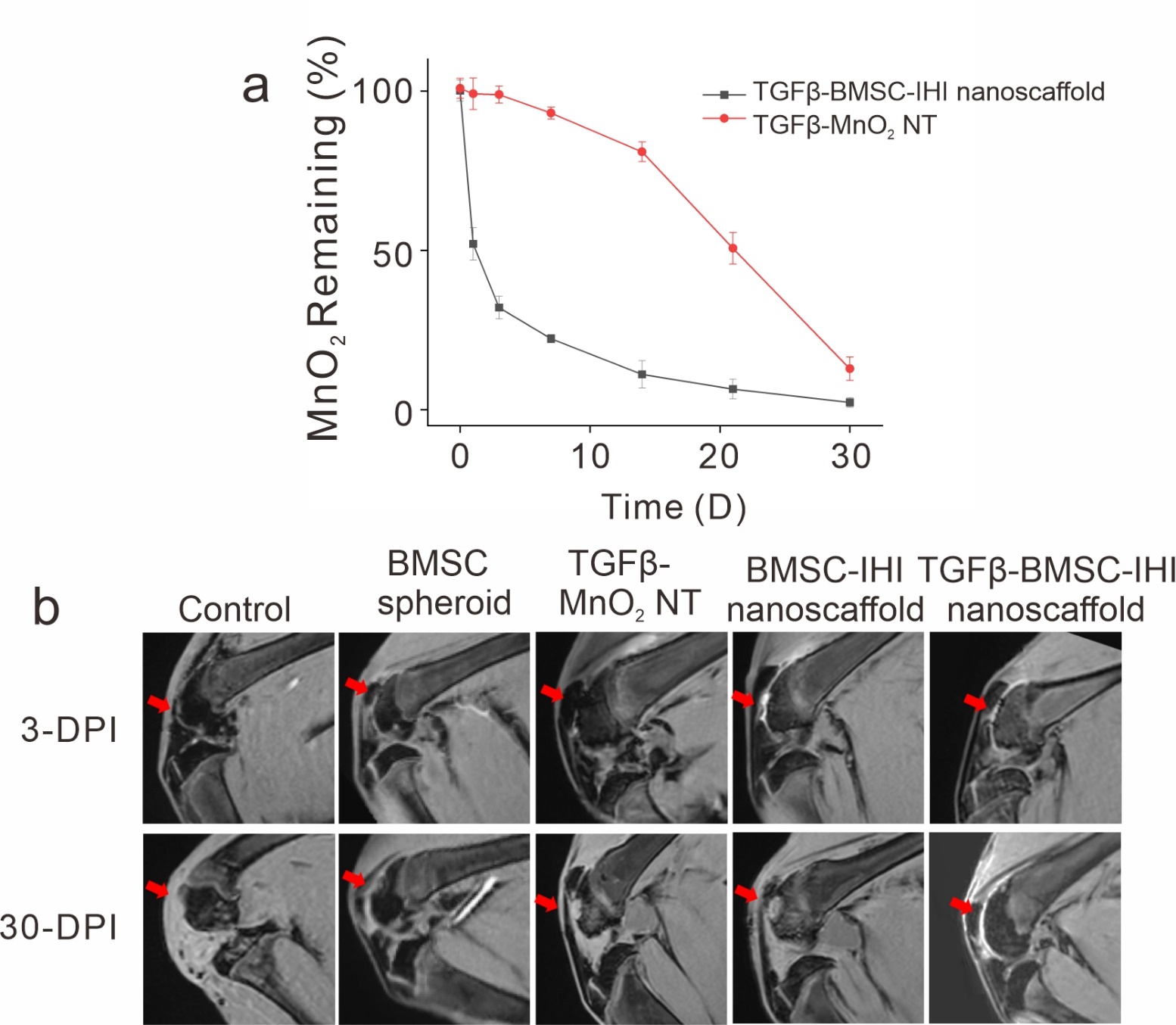


**Figure S19. *In vivo* degradation and MRI-monitorable disease progression.** a) The *in vivo* degradation of MnO_2_ nanotubes was calculated based on ICP-MS assay. b) Time-dependent (3-DPI, 30-DPI) MR imaging illustrating the biodegradation *in vivo* and the monitoring of cartilage regeneration process based on the MRI active Mn^2+^ ions. All data are presented as mean ± SD (n=3).


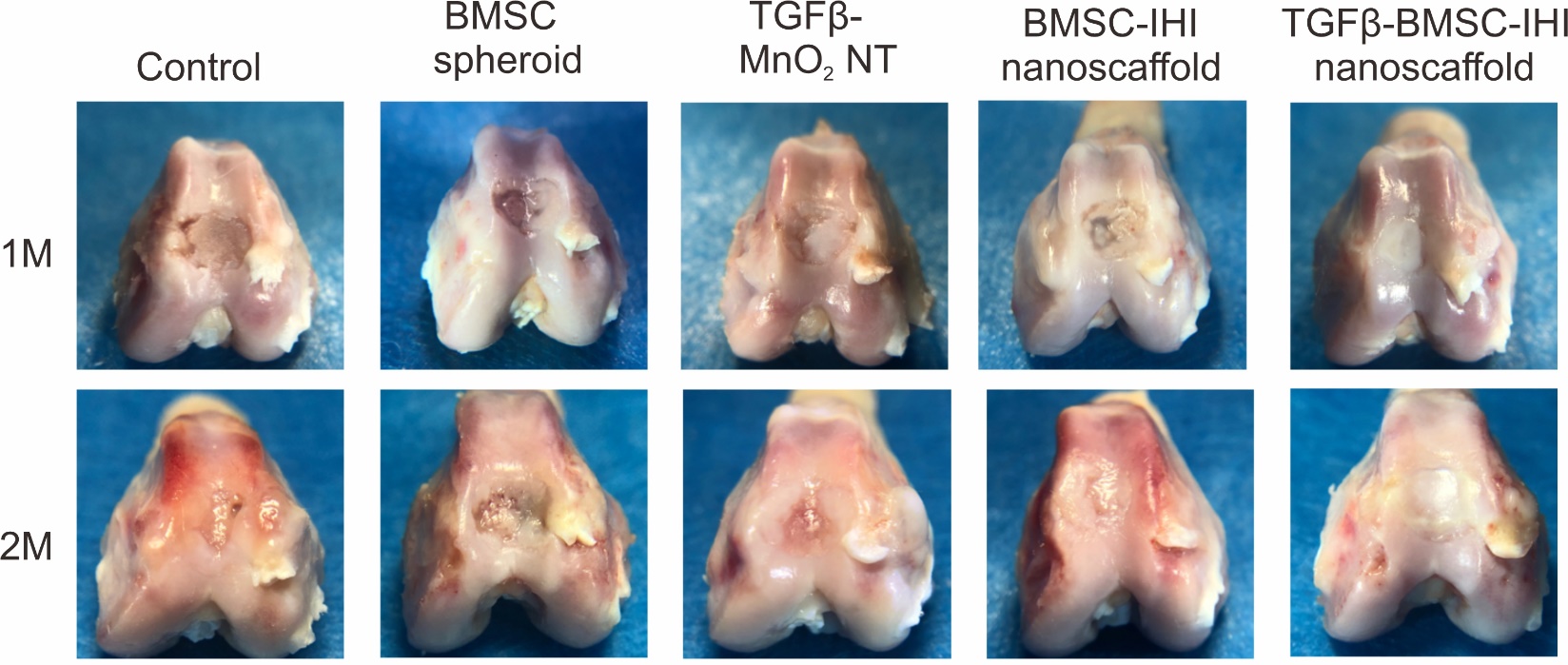


**Figure S20. Long-term cartilage regeneration.** Macroscopic images of cartilage tissues harvested at the three time points (1-MPI and 2-MP) were analyzed with their structural integrity.


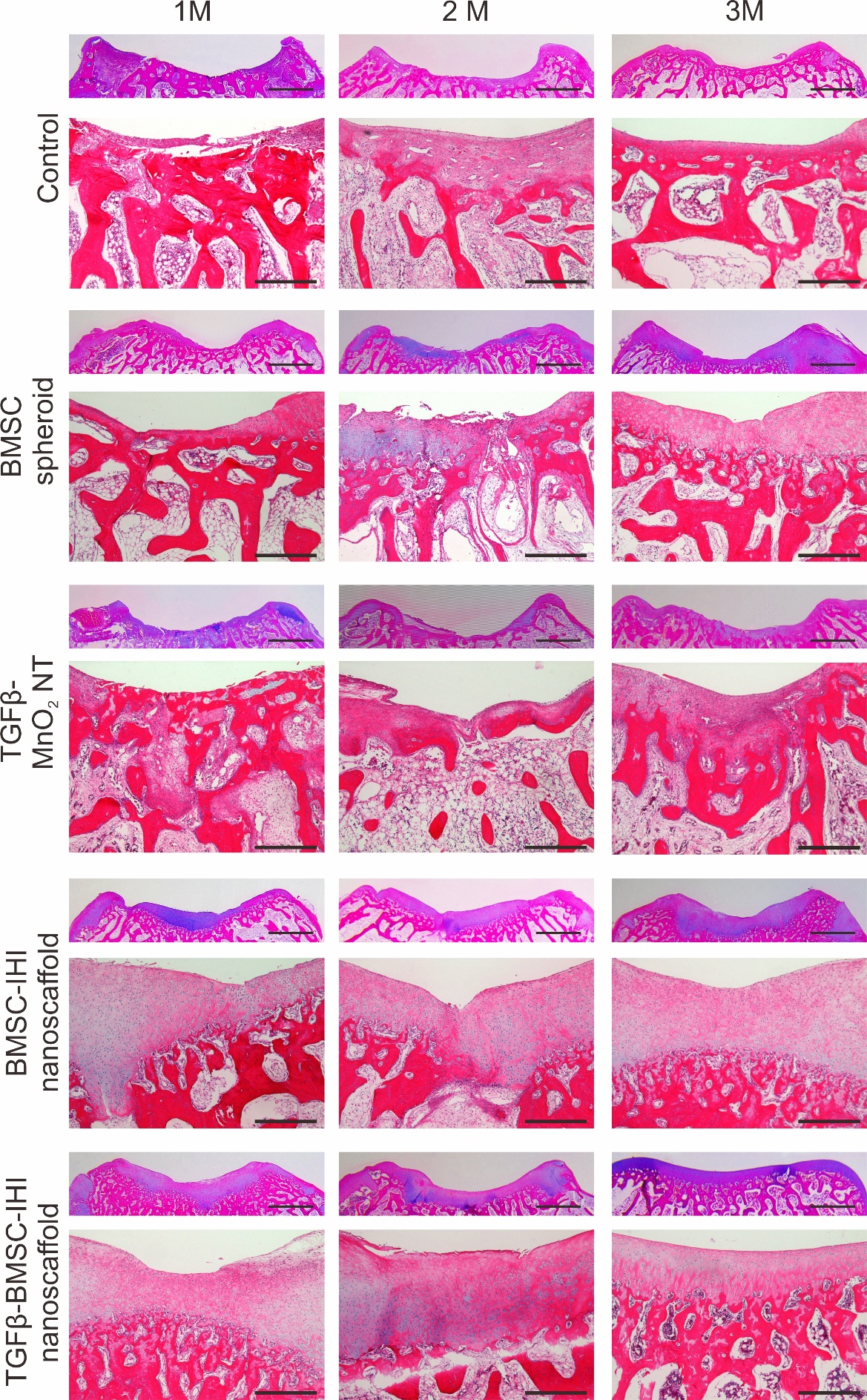


**SFigure S21. Enhancing *in vivo* cartilage regeneration using 3D-IHI nanoscaffold.** The regenerated cartilage tissues were characterized by Hematoxylin and Eosin (H&E) staining. Zoom out scale bar: 2 mm, Zoom in scale bar: 200 μm.


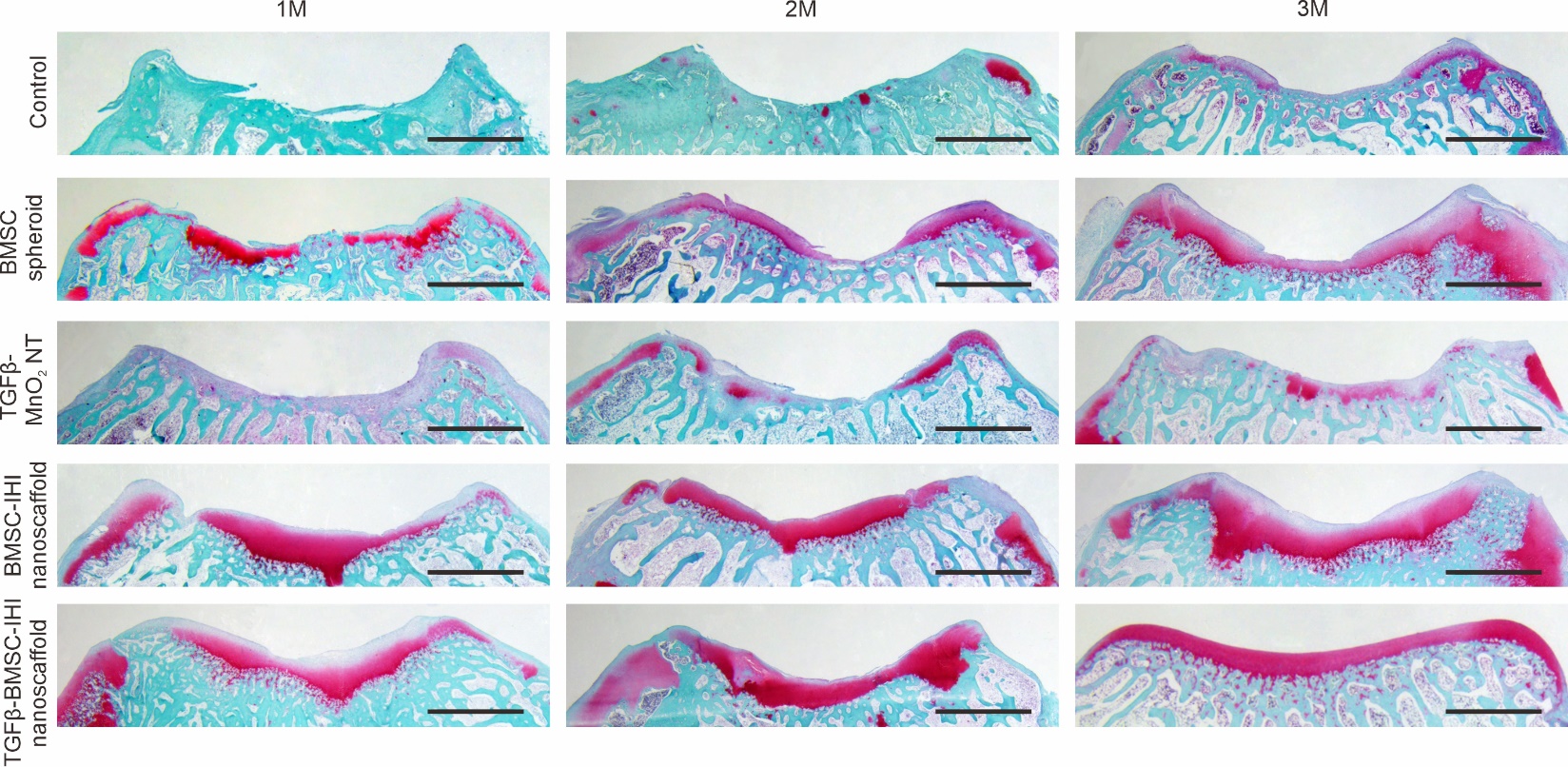


**Figure S22. Enhanced long-term cartilage regeneration after transplantation.** The long-term *in vivo* cartilage regeneration was characterized through Safranin O staining. A smooth and nearly complete structural recovery of the injured cartilage tissue with a higher population of chondrocytes in the experimental group (3D TGFβ-BMSC-IHI nanoscaffold) was observed on 3-MPI. Scale bar: 2 mm.


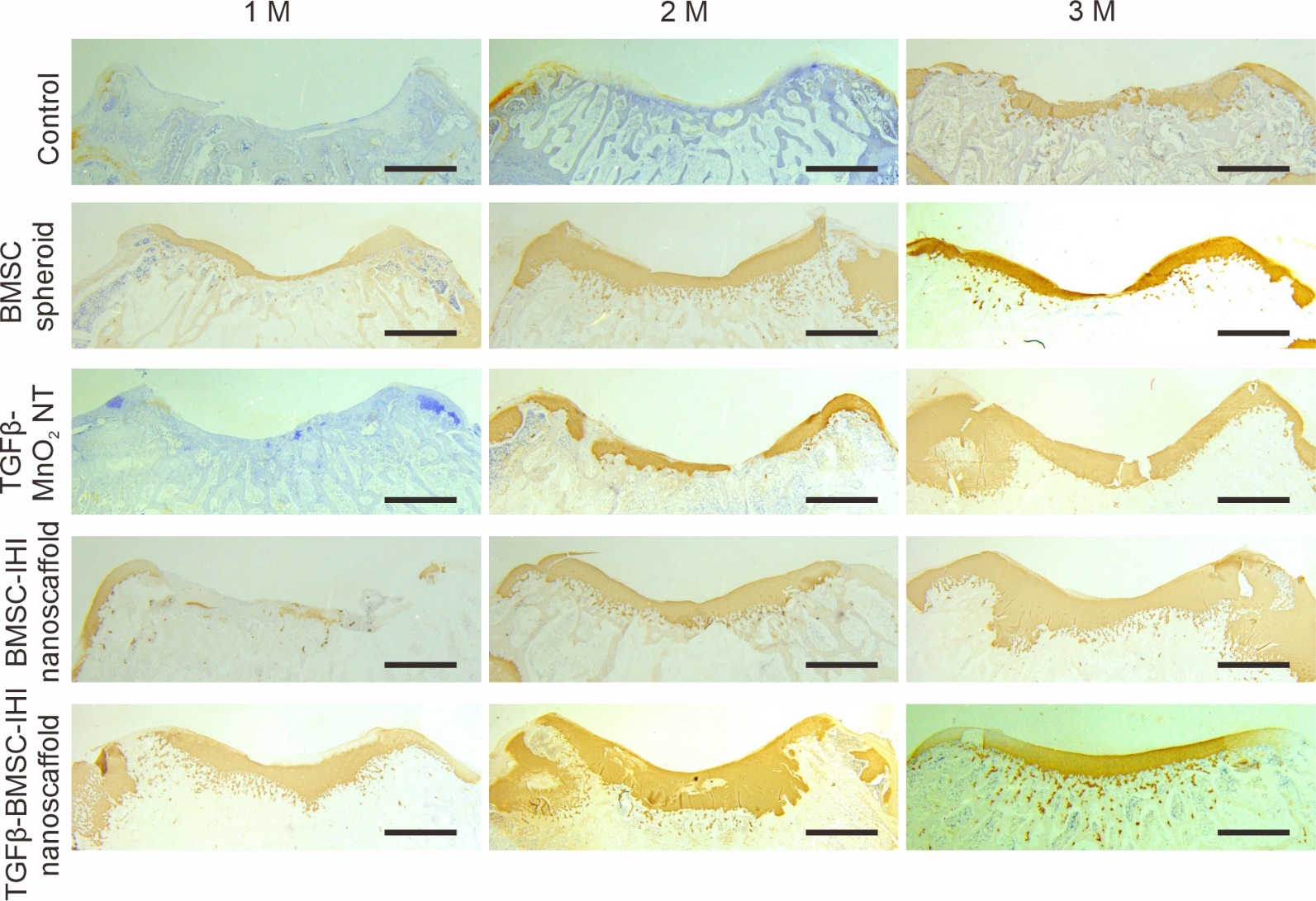


**Figure S23. Enhanced long-term cartilage regeneration via depositing ECM components after transplantation.** The long-term *in vivo* cartilage regeneration was characterized through Collagen-II (Col II) immunochemistry staining. Upregulated deposition of chondrogenic ECMs at the injury sites in the experimental group animals across all three time points indicate the enhanced cartilage regenerability of our 3D TGFβ-BMSC-IHI nanoscaffold. Scale bar: 2 mm.


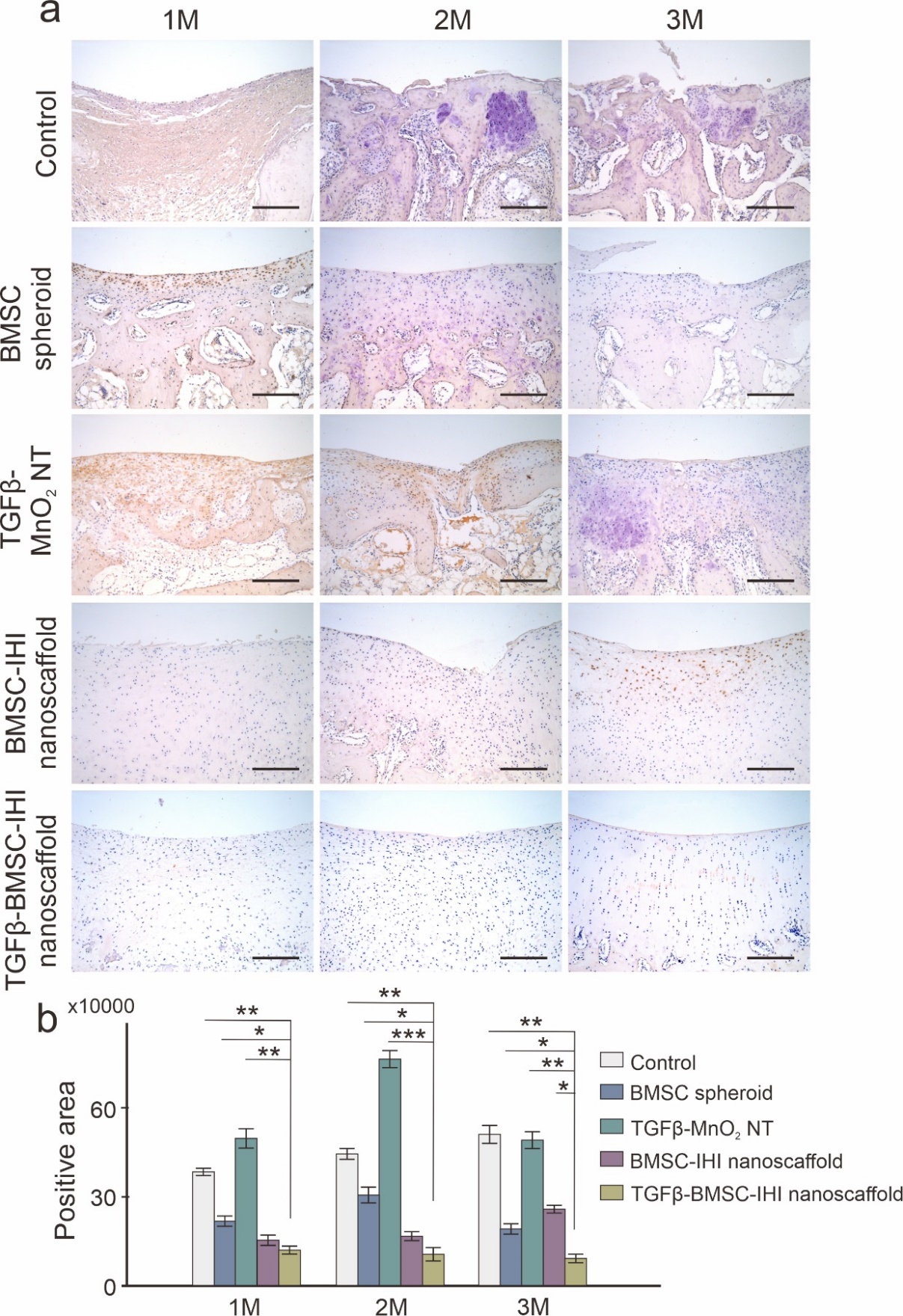


**Figure S24. Reduced uncontrollable differentiation of BMSC *in vivo*.** a-b) Type X collagen (Col X) immunostaining images (a) and the corresponding quantitative analysis (b) in the cartilage tissue slices. Scale bar: 200 μm. Our MnO_2_ nanotube templated stem cell assembly and homogeneous delivery of TGF-β3 could significantly decreased hypertrophic chondrocytes. All data are presented as mean ± SD (n=5). *p < 0.05, **p < 0.01, ***p < 0.001.


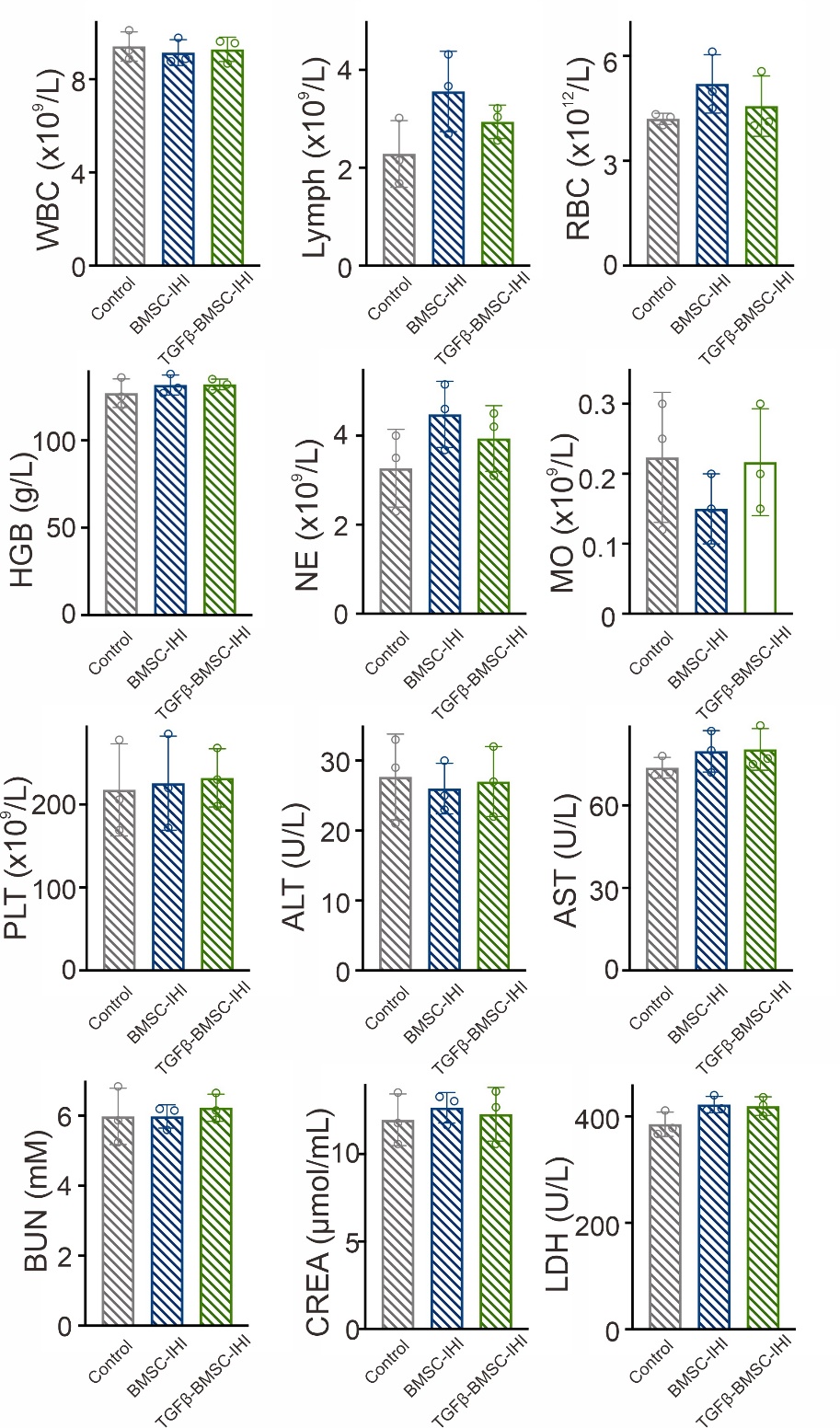


**Figure S25. *In vivo* compatibility assay of our 3D-IHI nanoscaffold.** The whole blood analysis and the liver, kidney and myocardium function analysis of the blood from the rabbits treated with 3D BMSC-IHI nanoscaffold and 3D TGFβ-BMSC-IHI nanoscaffold for 7 days. The levels of white blood cell (WBC), lymphocyte (Lymph), red blood cell (RBC), hemoglobin (HGB), neutrophils (NE), monocyte (MO) and platelets (PLT) were in the normal ranges, indicating there was no acute toxicity of the MnO_2_ nanotubes *in vivo*. The levels of liver damage indicators of alanine aminotransferase (ALT) and aspartate aminotransferase (AST), kidney damage indicator of blood urea nitrogen (BUN) and creatinine (CREA), as well as myocardium damage indicator of lactate dehydrogenase (LDH) were in the normal ranges, indicating the safety of MnO_2_ nanotubes to liver, kidney and heart *in vivo*. All data are presented as mean ± SD (n=3).


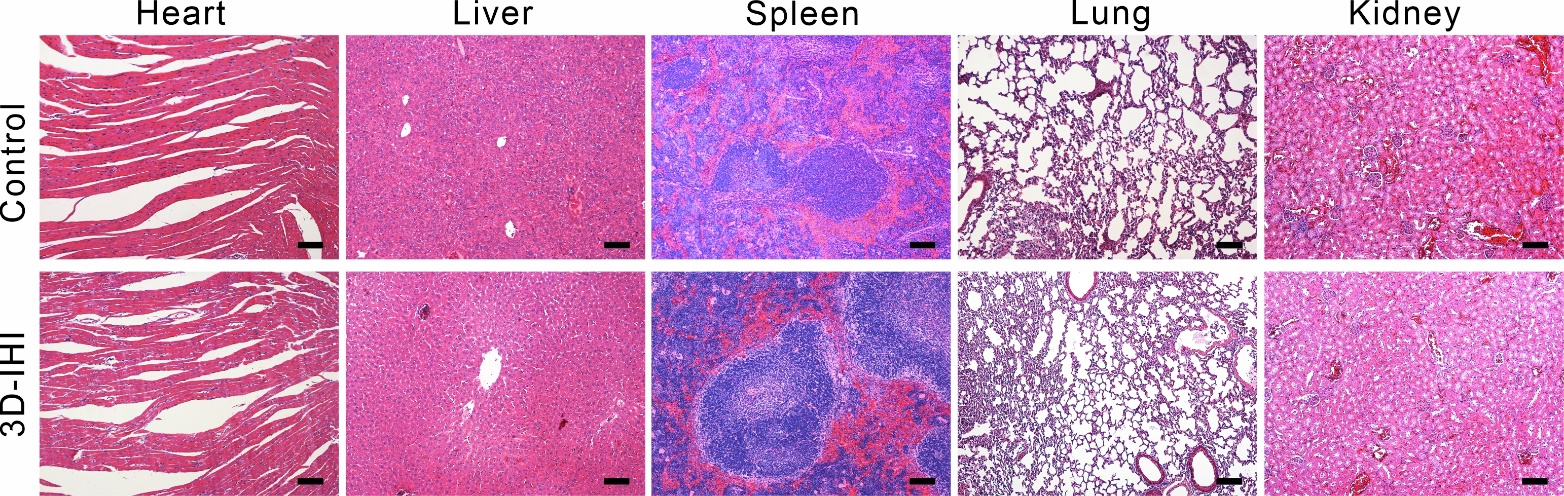


**Figure S26. Histomorphological evaluation of the main organs.** No noticeable toxicity was observed.


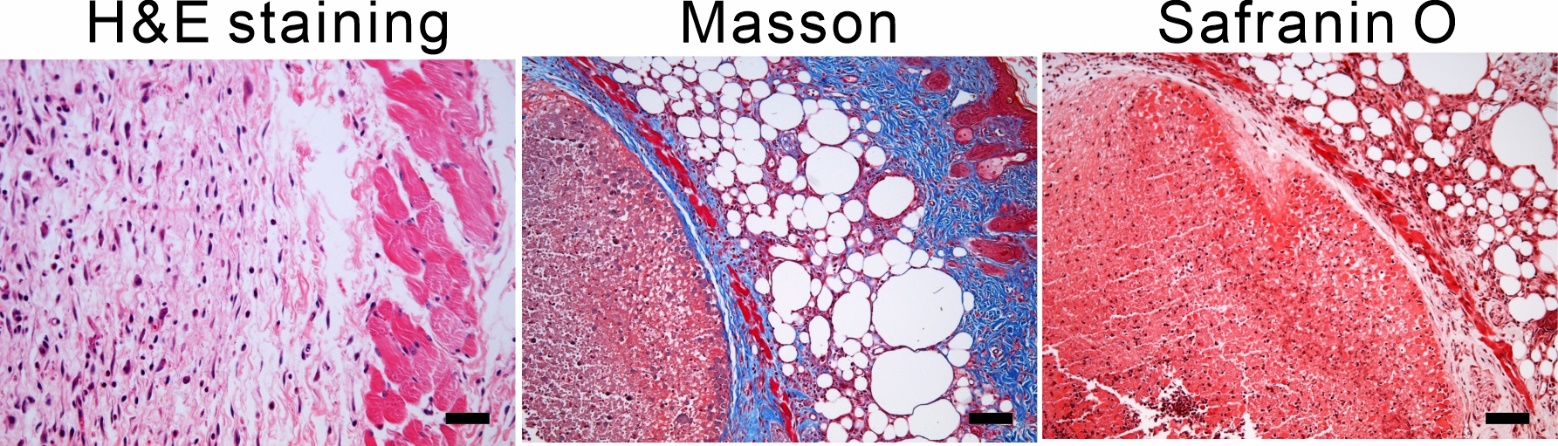


**Figure S27. Histomorphological evaluation of the skin.**


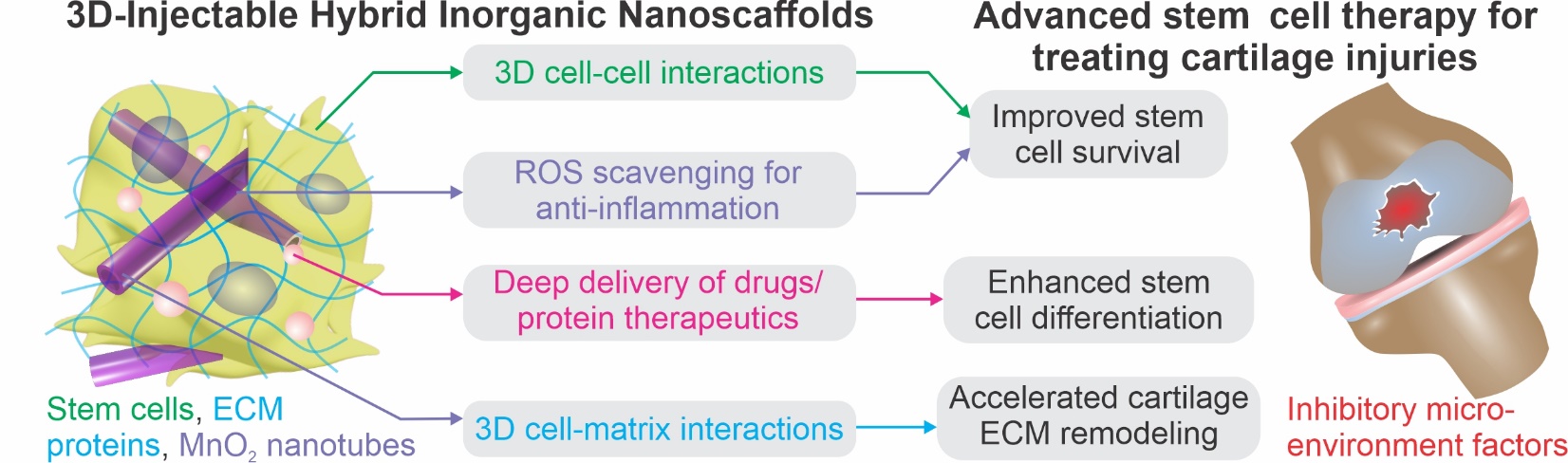


**Figure 28. Summary of the development of 3D-IHI nanoscaffold-based treatment of cartilage injuries.** 3D-IHI nanoscaffold that can be rapidly self-assembled from stem cells, ECM proteins, and MnO_2_ nanomaterials promote 3D cell-cell interactions, 3D cell-matrix interactions, and provides deep delivery of protein drugs (TGF-β3) as well as scavenges ROS for reduced inflammation. In this way, survival, chondrogenic differentiation of stem cells, and remodeling of cartilage ECM can be improved, thereby leading to functional recovery of cartilage injuries in the long term.

**Supplementary Table 1.** **Summary of terminologies and experimental conditions used for *in vitro* and *in vivo* experiments.**


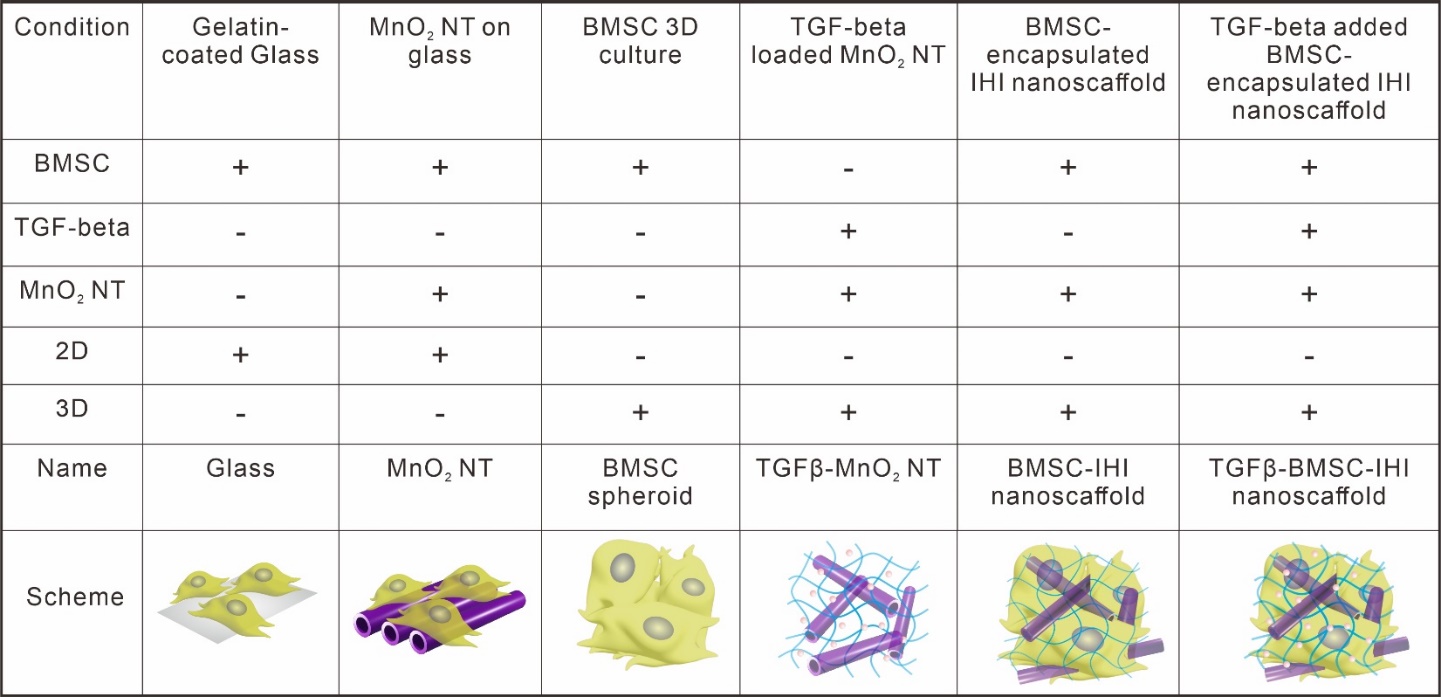


**Supplementary Table 2.** **The primer sequence for the genes which are analyzed.**

| **Gene** | **Forward Primer** | **Reverse Primer** |
| --- | --- | --- |
| GAPDH | CCGCATCTTCTTTTGCGTCG | GCCCAATACGACCAAATCCGT |
| SOX 9 | CTTCCGCGACGTGGACAT | GTTGGGCGGCAGGTACTG |
| Aggrecan | CTAGTGGACTCCCTTCAGGAAC | CGCTAAGCTCAGTCACTCCAG |
| Col II | ACCCTGAGTGGAAGAGTGGAG | CTTGGGAACGTTTGCTGGATTG |
| N-cadherin | CTGCAGAAAATCAAGTGCCA | ATGATCTTAGGATTGGGGGC |
| GSK-3β | CAGCAGCCTTCAGCTTTGG | CCGGAACATAGTCCAGCACCAG |
| β-catenin | AAGTTCTTGGCTATTACGACA | ACAGCACCTTCAGCACTCT |
| Integrin β1 | CTCCATCCTGGCCTCGCTGT | GCTGTCACCTTCACCGTTCC |
| FAK | TGGTGCAATGGAGCGAGTATT | CAGTGAACCTCCTCTGACCG |
| ERK1/2 | AATCACACGGTAGACACTGAAATGCC | CATCATCCCATCTAAAATGTCCCCTG |
